# Changes in cyanobacterial growth and photosynthetic pigment under different conditions

**DOI:** 10.1128/spectrum.03782-25

**Published:** 2026-06-15

**Authors:** Mohsen Seyedabadi, Arehzoo Zaker, Fatemeh Keykha Akhar, Maryam Ameri

**Affiliations:** 1 Razi Vaccine and Serum Research Institute, Agriculture Research, Education and Extension Organization (AREEO) 48405, Mashhad, Iran; 2 Department of Biology Education, Farhangian University 529365, Tehran, Iran; 3 Department of Plant Production and Genetics (Biotechnology), Faculty of Agriculture, Jahrom University 545281, Jahrom, Iran; 4 Department of Industrial Microbial Biotechnology, Research Institute for Industrial Biotechnology, Academic Center for Education, Culture, and Research (ACECR) 438298, Mashhad, Khorasan Razavi Province, Iran; Universidade do Porto Centro Interdisciplinar de Investigacao Marinha e Ambiental, Matosinhos, Portugal; East China Normal University, Shanghai, China

**Keywords:** *Calothrix*, cyanobacteria, *Microchaete*, phycobiliprotein, pigment, stress

## Abstract

Today, cyanobacteria, as the first photosynthetic microorganisms, play an undeniable role in ecosystems and global markets. In this research, the biomass and photosynthetic pigment production under environmental and nutritional factors in *Calothrix* and *Microchaete* were investigated. Photoperiod did not influence biomass production, while both strains produced maximum biomass at a light intensity of 100 µmol photons m^−2^ s^−1^. Although 100 mM NaCl and 25 mM NaNO_3_ increased the biomass in both cyanobacteria, urea at 1 mM inhibited the growth of *Calothrix*, in contrast to *Microchaete*. On the other hand, exposure to 10 mM hydrogen peroxide (H_2_O_2_) reduced *Microchaete* growth but did not affect *Calothrix* biomass. Both strains exhibited a significant increase in chlorophyll and carotenoid content under 16 h (in *Microchaete*) and 8 h (in *Calothrix*) of light exposure. Elevated NaCl concentrations (100 mM) and lower urea levels (1 mM) stimulated chlorophyll and carotenoid synthesis, while a decrease was observed under H_2_O_2_ treatment. The highest concentrations of total phycobiliproteins were observed at 10 and 50 µmol photons m^−2^ s^−1^ for *Calothrix* and *Microchaete*, respectively. While photoperiod changes did not affect total phycobiliprotein production in *Microchaete*, the optimal photoperiod for *Calothrix* was 8 h. Optimal phycobiliprotein production in *Calothrix* was achieved with 50 mM NaCl, 5 mM NaNO_3_, and 1 mM urea, whereas *Microchaete* responded best to 50 mM NaCl. At 10 mM H_2_O_2_, the total phycobiliprotein level in *Microchaete* remained unchanged, while *Calothrix* showed decreased total phycobiliproteins. Overall, *Calothrix* demonstrated superior production of phycobiliproteins, including phycoerythrin and phycocyanin, compared to *Microchaete*, highlighting its potential for pigment-based biotechnological applications.

**IMPORTANCE:**

This research provides critical insights into the metabolic flexibility of two cyanobacteria by investigating the effect of key environmental factors—light, salinity, nitrogen source, and induced oxidative stress—on the composition of photosynthetic pigment and biomass. The observed dynamic changes in photosynthetic metabolites, especially phycobiliproteins, reveal the interesting regulatory mechanisms these strains employ for photoacclimation and stress tolerance. Understanding these metabolite relationships is important for biotechnological applications. It allows for the optimized production of valuable natural pigments and the enhancement of biomass for biofertilizers. Furthermore, the study establishes a basic model for manipulating culture conditions to direct metabolic flux toward desired compounds, offering a strategic framework for further research on microalgae and other valuable microorganisms.

## INTRODUCTION

Cyanobacteria are ancient prokaryotic organisms that play a crucial role in oxygenic photosynthesis and biogeochemical cycles. They contribute substantially to photosynthetic biomass production and the global food cycle, accounting for approximately 25% of ocean primary productivity, particularly in central oceans, while serving as significant CO_2_ sinks (1, 2). Their nitrogen-fixing ability positions them as essential contributors to bioavailable nitrogen in marine ecosystems. These organisms thrive in various natural and artificial environments and are known for producing chemically unique secondary metabolites (3, 4). They are rich in proteins, polysaccharides, lipids, phycobiliproteins (PBPs), carotenoids, and chlorophylls, making them valuable sources of natural pigments and bioactive compounds (5). Under varying conditions of nutrient availability, light intensity and wavelength, temperature, and pH, cyanobacteria can modulate their metabolic activities and PBP composition via differential gene expression, particularly under stress conditions (6, 7). The growing demand for biologically active natural products as sustainable alternatives to synthetic compounds has intensified research into cyanobacteria. Their potential applications span multiple fields, including nutrition, medicine, wastewater treatment, renewable energy, and the chemical and pharmaceutical industries (7).

One of the most valuable compounds derived from cyanobacteria is PBPs, which are water-soluble, light-harvesting pigment proteins with broad industrial applications, including food, cosmetics, nutraceuticals, pharmaceuticals, biomedical research, clinical diagnostics, and animal feed (5, 7). Among PBPs, phycocyanin (PC) and phycoerythrin (PE) are especially promising, serving as natural colorants and bioactive agents with diverse health benefits. These include immunomodulatory, antioxidant, antiviral, anti-inflammatory, anticancer, anti-hepatitis, and cholesterol-lowering properties. Their fluorescent characteristics also make them valuable in molecular biology and therapeutic applications (8, 9). In addition, cyanobacterial chlorophylls and carotenoids exhibit potent antioxidant activity, capable of preventing lipid peroxidation, DNA damage, and oxidative stress-induced mitochondrial dysfunction. These metabolites are also utilized as edible pigments in food products (10, 11).

Abiotic factors such as light quality and intensity, temperature, nutrient levels, pH, and salinity can alter the physiological responses in cyanobacteria (7, 9). For commercial-scale production, optimizing culture conditions is essential to maximize biomass yield and compound synthesis (12–14). Salinity is a critical abiotic stress factor. High salt concentrations reduce water potential and increase ionic toxicity, thereby impairing cellular metabolism (15, 16). Efficient light utilization is also crucial, as specific light conditions affect the growth, photosynthetic activity, and biosynthesis of valuable compounds (9, 12, 17, 18). Controlled illumination systems that optimize intensity, spectral composition, and light-dark cycles have been suggested to enhance productivity while minimizing energy consumption (19). Cyanobacteria also respond to oxidative stress caused by reactive oxygen species (ROS), such as hydrogen peroxide (H_₂_O_₂_), by activating protective mechanisms, including the production of antioxidant metabolites. Given the antioxidant properties of PBPs, particularly PE and PC, oxidative stress may stimulate their accumulation, thereby enhancing their biotechnological value (20, 21).

*Calothrix* and *Microchaete* are filamentous cyanobacteria widely distributed in diverse habitats, including marine and freshwater environments, rice fields, and saline soils (22). *Calothrix* possesses the ability to fix atmospheric nitrogen, establish symbiotic associations, and has simpler nutritional requirements compared to other cyanobacterial genera (23, 24). *Calothrix* sp. 1SC65 holds considerable biotechnological potential due to its capacity to produce bioactive compounds, including pigments, enzymes, and pharmacologically active toxins. Its adaptability to various environmental conditions makes *Calothrix* a valuable candidate for natural product discovery and biotechnological applications (4). *Microchaete* sp. CCU342 is distinguished by its ability to produce PBPs, particularly PE, which are of high commercial and biomedical value (25). Despite their promising potential, research on how environmental factors like salinity, light intensity, and oxidative agents (e.g., H_2_O_2_) affect the growth and pigment production of these cyanobacteria remains limited. Manchanda and Sharma (26) found that increasing salinity (EC, 0.2–15 dS/m) in *Calothrix marchica* reduced the growth but enhanced lipid content. Ghadai et al. (27) observed that increasing salinity to 25% led to a decline in chlorophyll, carotenoid, and PC content in *Calothrix juliana*. Nitrogen-dependent variation in carotenoid composition of *Calothrix* was demonstrated by Kosourov et al. (28). The effect of light intensity on the production of volatile organic iodine substances in *Calothrix parasitica* was also studied by irradiating the culture medium (29). In *Microchaete*, Hemlata and Fatma (25) identified strain CCU342 as a good source for PE production. However, Thajamanbi et al. (22) reported that *Microchaete* and *Calothrix* isolated from rice fields exhibited relatively low PE levels compared to other pigments. Notably, *Microchaete* demonstrated higher levels of total carotenoids (3.11 μg mL^−1^), whereas *Calothrix* accumulated more chlorophyll *a* (13.84 μg mL^-1^) and PBPs (103.35 mg g^−1^), highlighting species-specific pigment profiles.

Given the importance of optimizing culture conditions to enhance biomass and bioactive metabolite yields, the present study aims to investigate the growth and pigment production, including chlorophyll, carotenoids, total PBPs, PE, PC, and allophycocyanin (APC) of *Calothrix* and *Microchaete* under varying conditions of light intensity and concentrations of NaCl, NaNO_3_, urea, and H_2_O_2_.

## RESULTS

### The effect of light intensity and photoperiod on the growth and pigment profile

**Fig 1:**
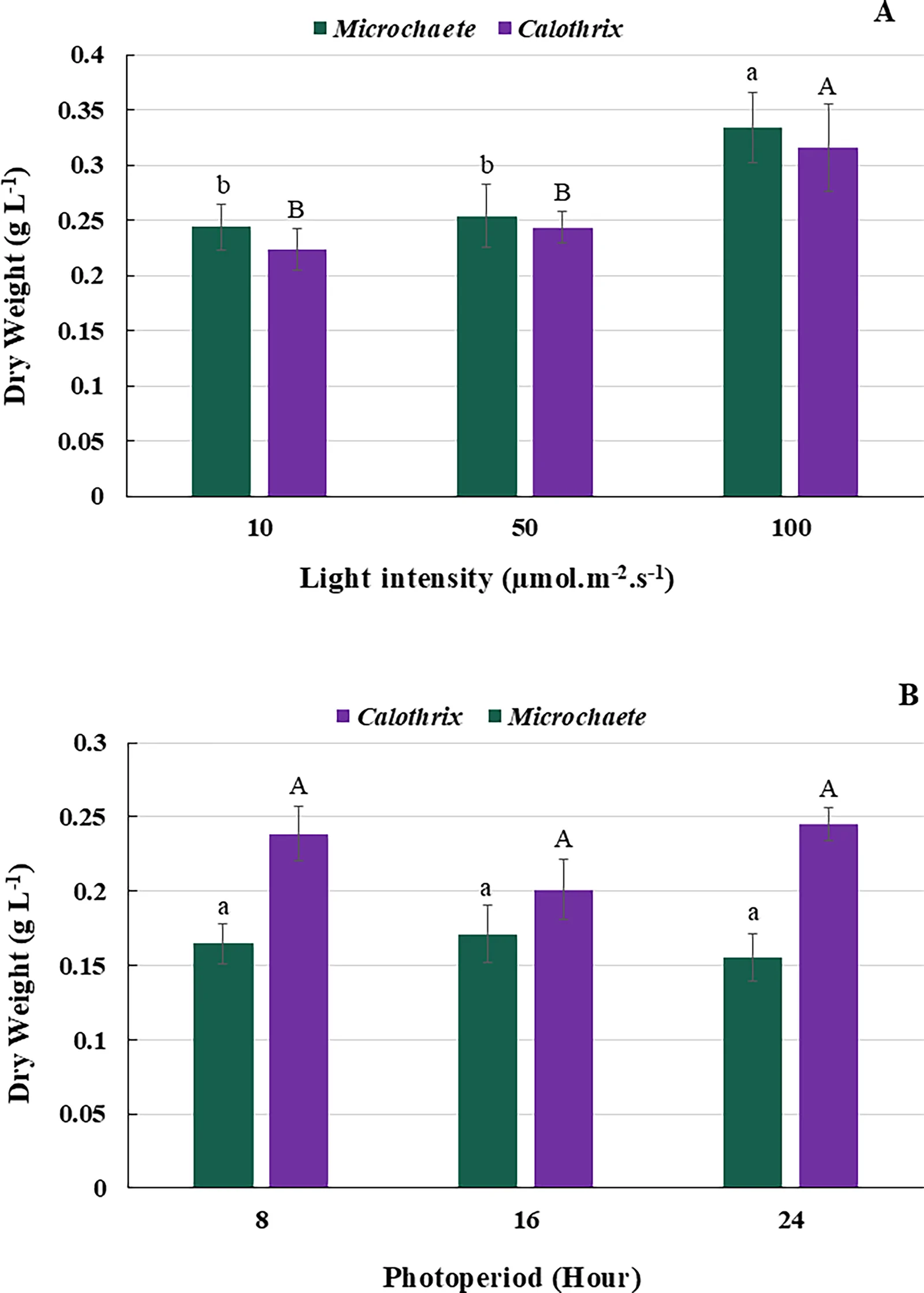
The effect of light intensity (**A**) and photoperiod (**B**) on biomass production in *Microchaete* and *Calothrix*. Data are mean values of three independent biological replicates. Different letters in each column indicate significant differences (*P* ≤ 0.05).

The results showed that both *Microchaete* and *Calothrix* achieved the highest biomass production at a light intensity of 100 µmol photons m^−2^ s^−1^ (Fig. 1A), reaching 0.33 and 0.31 g L^−1^ dry weight, respectively, after 2 weeks of cultivation. In contrast, the photoperiod did not significantly affect the growth of either cyanobacterium under the light intensity used (*P* > 0.05) (Fig. 1B). In other words, enhanced biomass production of *Calothrix* under a 24-h photoperiod (0.25 g L^-1^) and *Microchaete* under a 16-h photoperiod (0.17 g L^−1^) was not significantly different from their growth under other photoperiod regimes.

**Fig 2:**
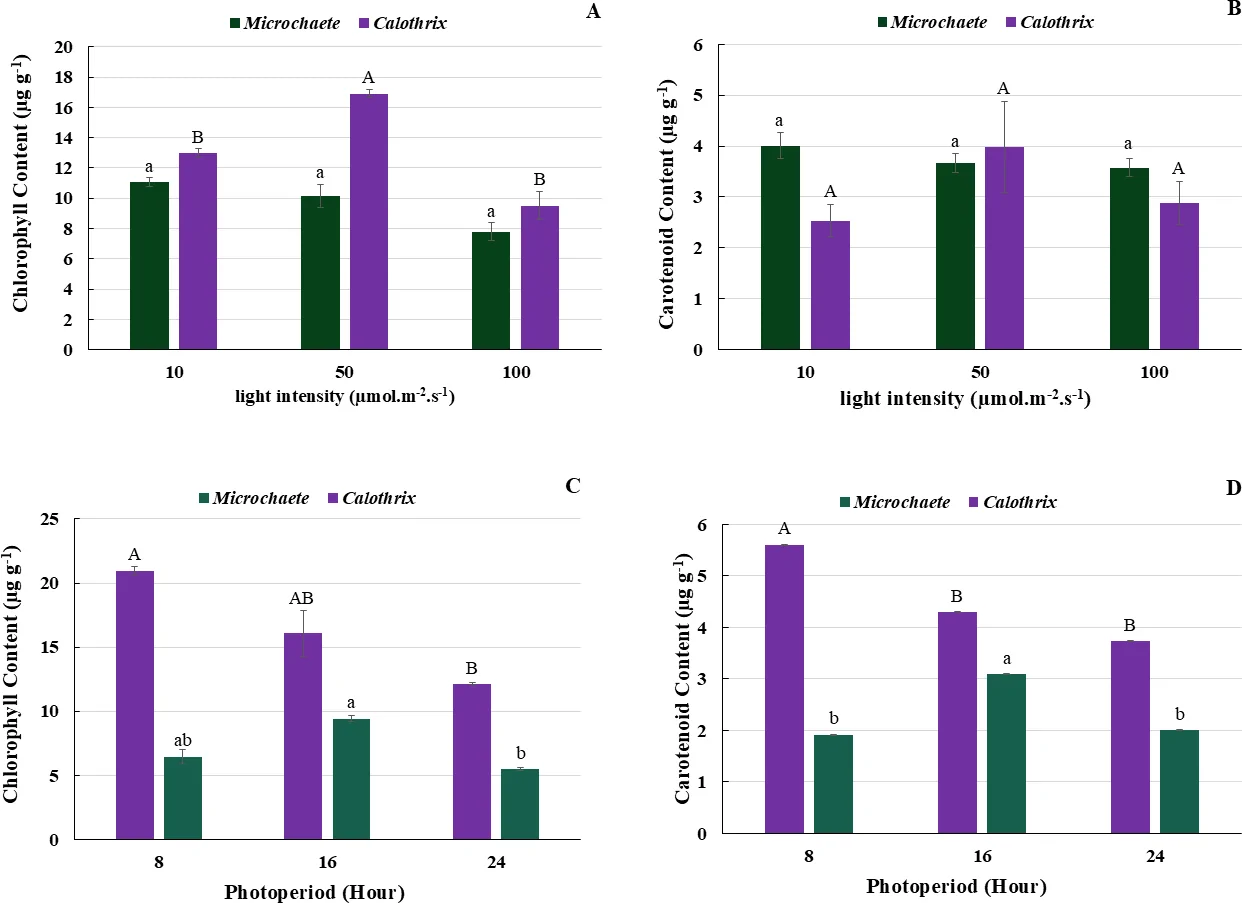
The effect of light intensity (**A and B**) and photoperiod (**C and D**) on chlorophyll and carotenoid content in *Microchaete* and *Calothrix*. Data are mean values of three independent biological replicates. Different letters in each column indicate significant differences (*P* ≤ 0.05).

According to the results, higher light intensities reduced the chlorophyll and carotenoid content in *Microchaete* (Fig. 2A and B), although the reduction was not statistically significant. The highest concentrations of chlorophyll (11.07 µg g^−1^) and carotenoid (4.01 µg g^−1^) in *Microchaete* were observed under 10 µmol photons m^−2^ s^−1^. In contrast, a light intensity of 50 µmol photons m^−2^ s^−1^ resulted in higher chlorophyll (16.91 µg g^−1^) and carotenoid (3.98 µg g^−1^) production in *Calothrix*, although variations in our light intensity did not significantly affect its carotenoid content. These findings suggest that higher illumination at 100 µmol photons m^−2^ s^−1^ is suitable to support the growth of both cyanobacteria.

As the duration of light exposure increased, chlorophyll and carotenoid levels in *Calothrix* decreased. The highest concentrations of these photosynthetic pigments were observed under 8 h of light exposure (20.96 and 5.60 µg g^−1^, respectively) (Fig. 2C and D). Conversely, *Microchaete* exhibited maximum chlorophyll (9.43 µg g^−1^) and carotenoid (3.09 µg g^−1^) production under 16 h of illumination.

**Fig 3:**
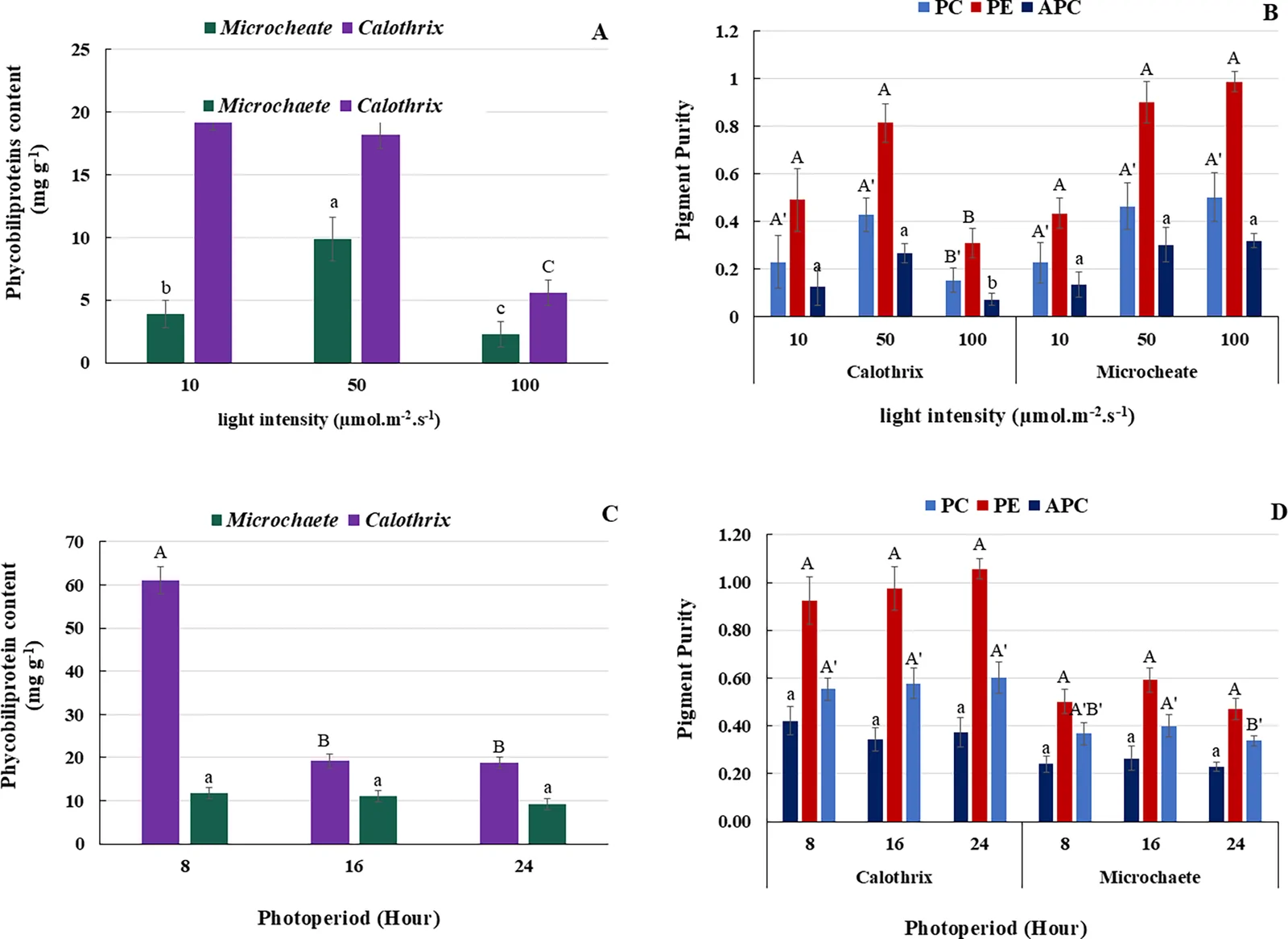
The effect of light intensity and photoperiod on total phycobiliprotein content (**A and B**) and purity of phycocyanin, phycoerythrin, and allophycocyanin (**C and D**) in *Microchaete* and *Calothrix*. Data are mean values of three independent biological replicates. Different letters in each column indicate significant differences (*P* ≤ 0.05).

Generally, more PBPs were produced in *Calothrix* compared to *Microchaete* under different light treatments. Among the tested light intensities, the highest total PBP content was observed in *Microchaete* (9.84 mg g^−1^) and *Calothrix* (20.15 mg g^−1^) under 50 and 10 µmol photons m^−2^ s^−1^ illumination, respectively (Fig. 3A). A higher light intensity (100 µmol m^−2^ s^−1^) significantly reduced the content of total PBPs in both cyanobacteria. While photoperiod had no significant effect on total PBP content in *Microchaete*, *Calothrix* exhibited maximum PBP production (60.99 mg g^−1^) under the shortest illumination duration (8 h) (Fig. 3C).

The purity of PE, PC, and APC in *Microchaete* was not affected by our light intensities (Fig. 3B). However, with increasing light intensities, the purity of these pigments in *Microchaete* improved. Cultures grown under 100 µmol photons m^−2^ s^−1^ illumination exhibited the highest purity of PE (0.99), PC (0.50), and APC (0.32). In contrast, the best purity of PE (0.81), PC (0.43), and APC (0.27) in *Calothrix* was observed at 50 µmol photons m^−2^ s^−1^, while the lowest pigment purity in this strain was recorded at 100 µmol photons m^−2^ s^−1^ (Fig. 3B).

No significant changes in the purity of PBPs in *Microchaete* were observed with variations in light exposure duration, except under 24-h illumination, which significantly decreased PC purity (Fig. 3D). In *Calothrix*, the observed changes in PE, PC, and APC purity across different photoperiods were not significant, while at higher light intensity (100 µmol photons m^−2^ s^−1^), they decreased. Overall, PE exhibited higher purity than PC and APC in both cyanobacterial strains.

**Fig 4:**
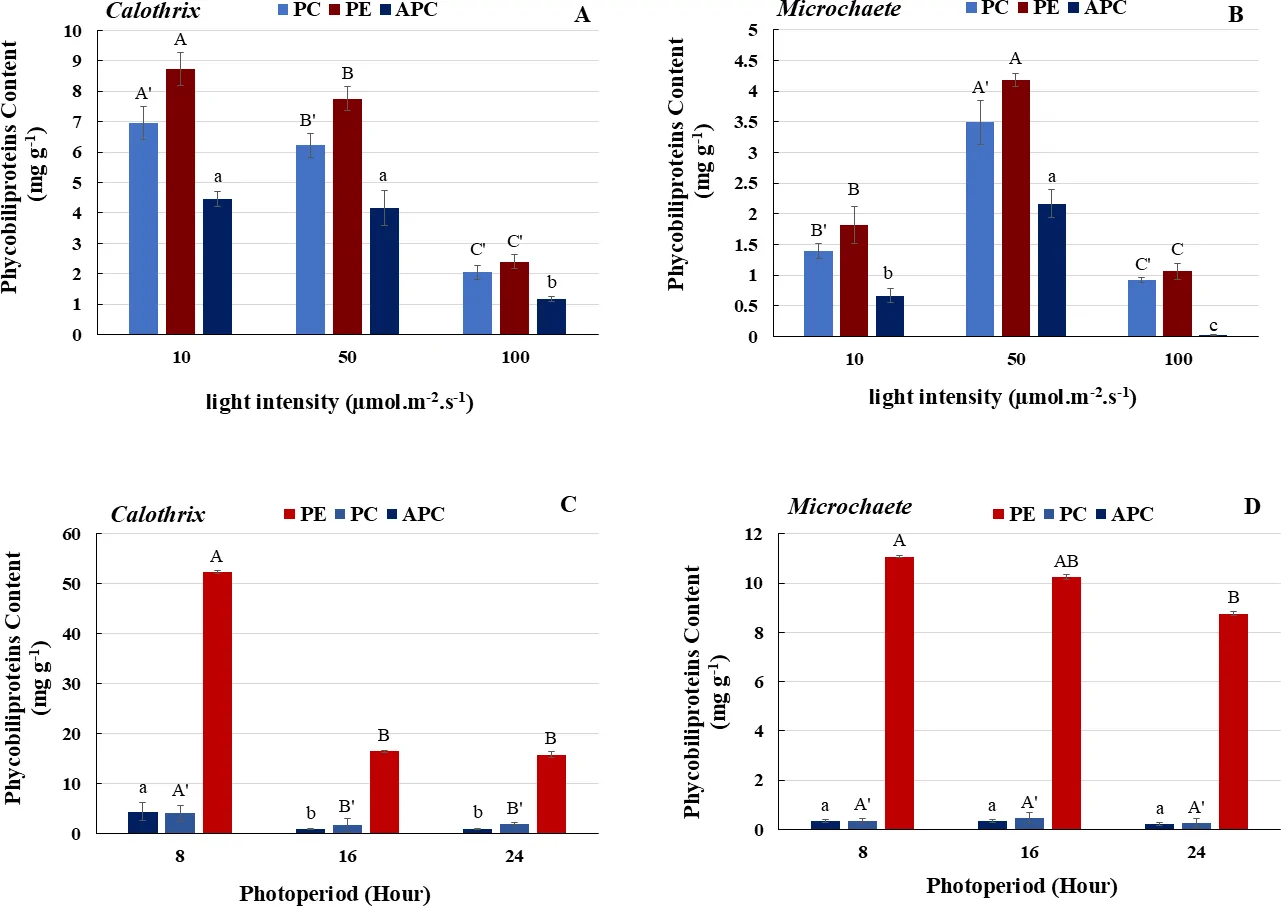
The effect of light intensity (**A and B**) and photoperiod (**C and D**) on the content of phycocyanin, phycoerythrin, and allophycocyanin in *Microchaete* and *Calothrix*. Data are mean values of three independent biological replicates. Different letters in each column indicate significant differences (*P* ≤ 0.05).

In both *Microchaete* and *Calothrix*, PE constituted the majority of PBPs (Fig. 4). Our high light intensities reduced the content of PE, PC, and APC in *Calothrix* (Fig. 4A). Exposure to 10 µmol photons m^−2^ s^−1^ led to significantly higher production of PE (8.73 mg g^−1^), PC (6.95 mg g^−1^), and APC (4.47 mg g^−1^) in this strain. In *Microchaete*, the highest pigment levels (4.18 mg g^−1^ for PE, 3.49 mg g^−1^ for PC, and 2.16 mg g^−1^ for APC) were observed at a light intensity of 50 µmol photons m^−2^ s^−1^ (Fig. 4B).

Increasing the duration of light exposure in both *Calothrix* and *Microchaete* led to a decrease in PE content (Fig. 4C and D). This trend was also evident for PC and APC in *Calothrix*. The highest PE concentrations in *Microchaete* (11.7 mg g^−1^) and *Calothrix* (52.34 mg g^−1^) were recorded under 8 h of light exposure at 50 µmol photons m^−2^ s^−1^. Photoperiod variation did not significantly change the content of PC and APC in *Microchaete* (Fig. 4D). Overall, PBP production in *Calothrix* was higher than in *Microchaete* across different light treatments.

### The effect of nutrients and salinity on the growth and pigment profile

**Fig 5:**
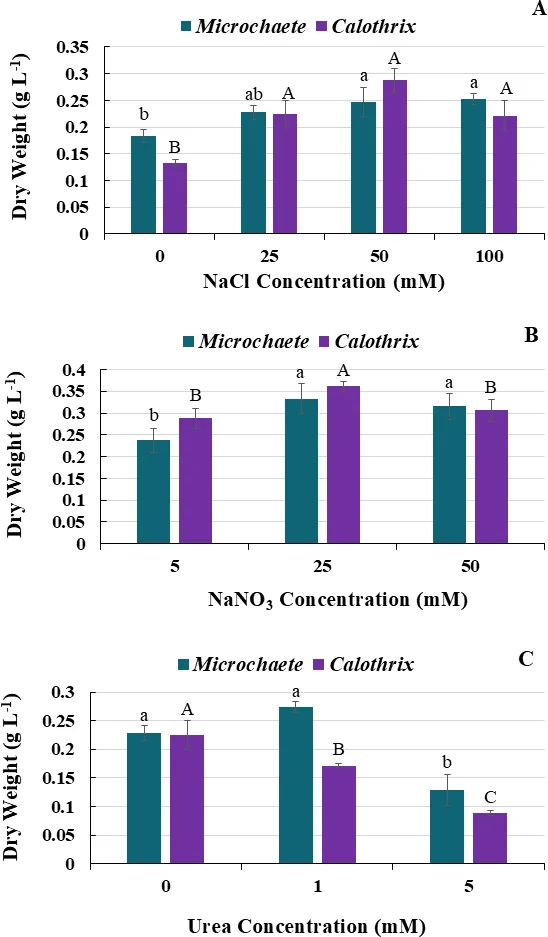
Biomass production in the presence of different concentrations of NaCl (**A**), NaNO_3_ (**B**), and urea (**C**) in *Microchaete* and *Calothrix*. Data are mean values of three independent biological replicates. Different letters indicate significant differences (*P* ≤ 0.05).

The presence of NaCl in the culture medium led to a significant increase in the dry weight of *Microchaete* and *Calothrix* compared to the control. *Calothrix* showed its highest biomass production (0.29 g L^−1^) at 50 mM NaCl, while *Microchaete* obtained maximum biomass (0.25 g L^−1^) at 100 mM NaCl (Fig. 5A).

Supplementation with 25 mM NaNO_3_ in the presence of 50 mM NaCl enhanced the growth of both cyanobacteria. Under these conditions, *Microchaete* and *Calothrix* achieved dry biomass yields of 0.33 and 0.36 g L^−1^, respectively. Although the growth of both cyanobacteria declined at 50 mM NaNO_3_, the reduction was not statistically significant compared to the 25 mM treatment in *Microchaete* (Fig. 5B). A low concentration of urea (1 mM) slightly stimulated the growth of *Microchaete*, yielding 0.27 g L^−1^ of biomass. However, increasing the urea concentration to 5 mM inhibited biomass production (Fig. 5C). In *Calothrix*, urea led to a significant decrease in biomass, resulting in a 1.7-fold reduction in dry weight at 5 mM urea (Fig. 5C).

**Fig 6:**
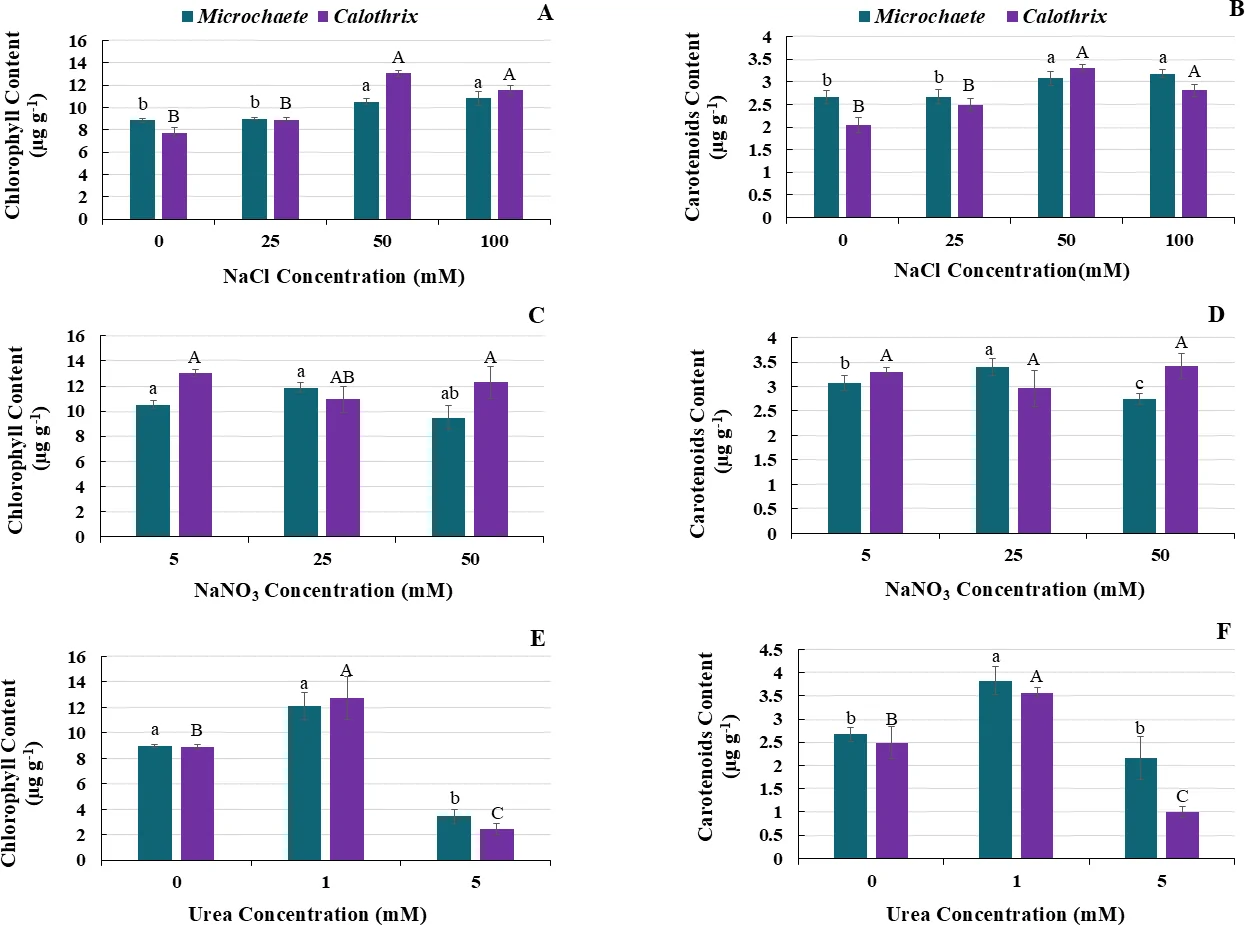
The effect of different concentrations of NaCl (**A and B**), NaNO_3_ (**C and D**), and urea (**E and F**) on chlorophyll and carotenoid content in *Microchaete* and *Calothrix*. Data are mean values of three independent biological replicates. Different letters indicate significant differences (*P* ≤ 0.05).

As observed in the growth patterns, salinity up to 100 mM NaCl promoted chlorophyll and carotenoid production (Fig. 6A and B). In both cyanobacteria, the increased chlorophyll and carotenoid content at 100 mM NaCl was not significant compared to that at 50 mM NaCl. At 50 mM NaCl, *Calothrix* exhibited higher chlorophyll and carotenoid levels than *Microchaete*. Despite the increased chlorophyll production in *Calothrix* (13.04 µg g^−1^) at 5 mM NaNO_3_ and in *Microchaete* (11.88 µg g^−1^) at 25 mM NaNO_3_, variations in chlorophyll content at different NaNO_3_ concentrations were not statistically significant in either cyanobacterium (Fig. 6C).

While changing NaNO_3_ levels had no significant effect on carotenoid content in *Calothrix*, the maximum production of this pigment in *Microchaete* (3.39 µg g^−1^) was observed at 25 mM NaNO_3_ (Fig. 6D). The results indicated that 1 mM urea effectively enhanced chlorophyll and carotenoid production in both tested cyanobacteria. However, at a higher concentration (5 mM), urea exhibited an inhibitory effect (Fig. 6E and F).

**Fig 7:**
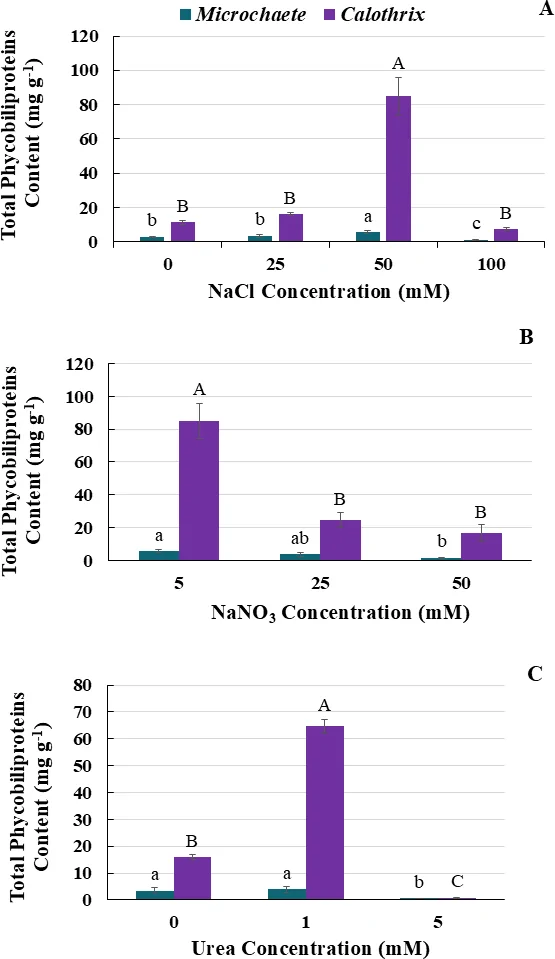
The effect of different concentrations of NaCl (**A**), NaNO_3_ (**B**), and urea (**C**) on total phycobiliprotein content in *Microchaete* and *Calothrix*. Data are mean values of three independent biological replicates. Different letters indicate significant differences (*P* ≤ 0.05).

*Calothrix* produced greater total PBP content than *Microchaete* at different concentrations of NaCl, NaNO_3_, and urea (Fig. 7). A concentration of 50 mM NaCl enhanced PBP production in *Calothrix* and *Microchaete* by 7.5- and 1.8-fold, respectively, compared to the controls (Fig. 7A). The presence of 5 mM NaNO_3_ stimulated the accumulation of PBPs in *Calothrix* to 85.16 mg L^−1^, which was 15.4 times higher than in *Microchaete* (Fig. 7B). However, higher levels of NaNO_3_ led to a decline in PBP content in both cyanobacteria (Fig. 7B). At a concentration of 1 mM urea, PBP levels in *Calothrix* and *Microchaete* reached 64.65 and 4.03 mg L^−1^, respectively (Fig. 7C). Under this treatment, the PBP content in *Calothrix* was four times higher than that of the control, whereas *Microchaete* showed no significant difference.

**Fig 8:**
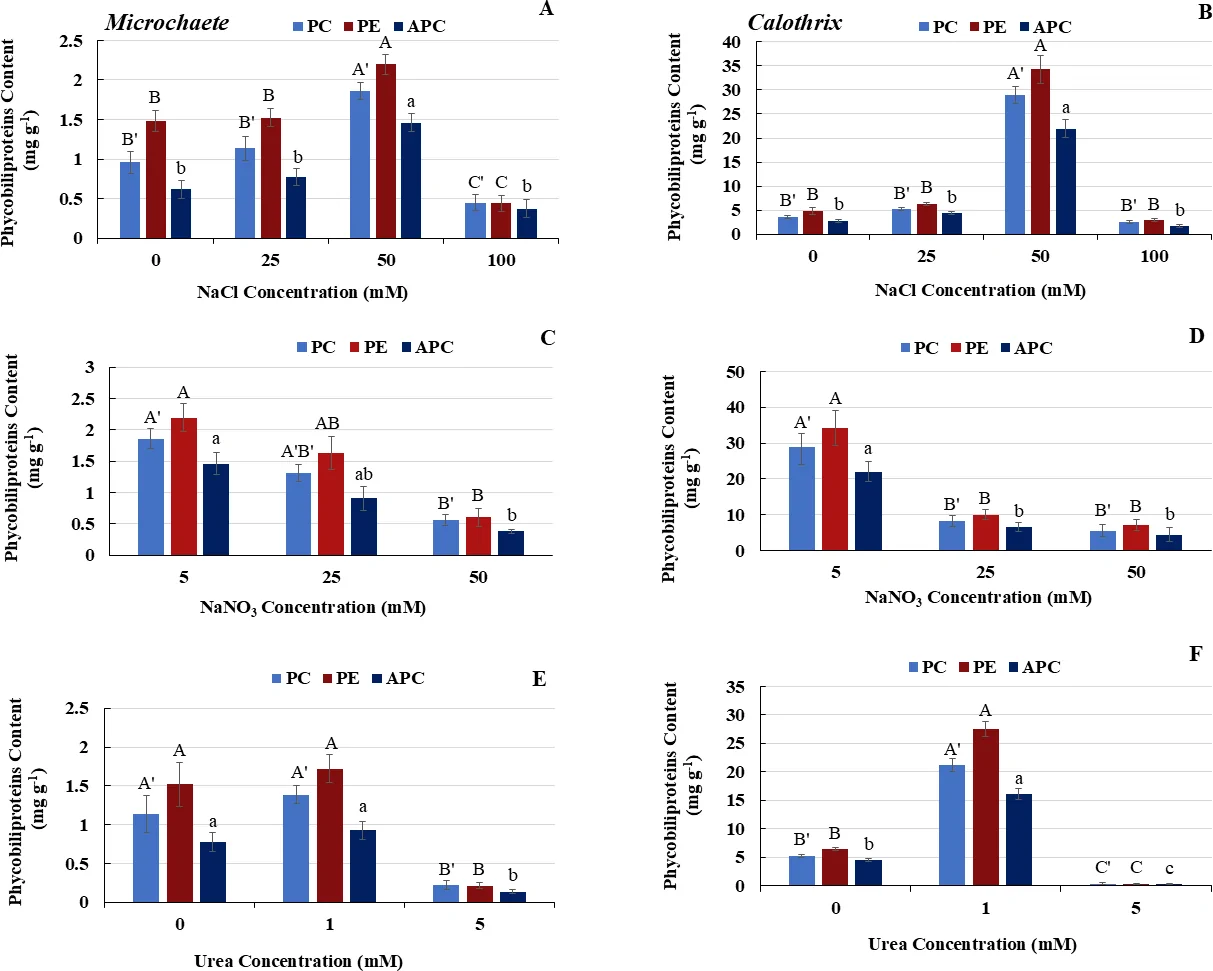
The effect of different concentrations of NaCl (**A and B**), NaNO_3_ (**C and D**), and urea (**E and F**) on the content of phycocyanin, phycoerythrin, and allophycocyanin in *Microchaete* (left) and *Calothrix* (right). Data are mean values of three independent biological replicates. Different letters indicate significant differences (*P* ≤ 0.05).

The results showed that treatment with 50 mM NaCl significantly enhanced PE, PC, and APC content in both *Microchaete* and *Calothrix* (Fig. 8A and B). The increased amount of these metabolites in *Calothrix* was more impressive than in *Microchaete*. In the presence of 50 mM NaCl, the concentrations of PE, PC, and APC in *Microchaete* were recorded as 2.19, 1.86, and 1.46 mg g^−1^, respectively. In *Calothrix*, these concentrations reached 34.27, 28.95, and 21.94 mg g^−1^, respectively. However, exposure to 100 mM NaCl resulted in a significant reduction in PE and PC content in *Microchaete* compared to the control. As shown in Fig. 8C and D, the PE, PC, and APC yields in both cyanobacteria gradually decreased with increasing NaNO_3_ concentration in the culture medium, with maximum production achieved at 5 mM NaNO_3_. Urea at a concentration of 1 mM had no significant effect on the content of PE, PC, and APC in *Microchaete* (Fig. 8E). However, in *Calothrix*, 1 mM urea significantly increased PE, PC, and APC concentrations by 4.3-, 4-, and 3.6-fold, respectively (Fig. 8F). Conversely, treatment with 5 mM urea led to a substantial reduction in these pigments. Overall, in both *Microchaete* and *Calothrix*, PE exhibited the highest PBP content, while APC showed the lowest.

In all nutritional treatments applied to *Microchaete* and *Calothrix*, PE exhibited the highest pigment purity (Table 1). In both cyanobacterial strains, the highest PE purity was observed under conditions of 50 mM NaCl and 5 mM NaNO_3_. While 1 mM urea enhanced PE purity in *Calothrix*, the maximum purity of this pigment in *Microchaete* was recorded in the absence of urea.

**TABLE 1:** The effect of different concentrations of NaCl, NaNO_3_, and urea on the purity of phycocyanin, phycoerythrin, and allophycocyanin in *Microchaete* and *Calothrix^a^^,^^b^*

Treatment	Concentration (mM)	*Microchaete*			*Calothrix*		
		PC	PE	APC	PC	PE	APC
NaCl	0	0.07 ± 0.010	0.15 ± 0.010	0.05 ± 0.007	0.34 ± 0.022	0.46 ± 0.033	0.29 ± 0.061
	25	0.08 ± 0.004	0.16 ± 0.008	0.05 ± 0.003	0.19 ± 0.010	0.35 ± 0.014	0.14 ± 0.011
	50	0.14 ± 0.010	**0.25** ± 0.014	0.08 ± 0.009	0.60 ± 0.080	**1.07** ± 0.120	0.42 ± 0.032
	100	0.07 ± 0.005	0.04 ± 0.005	0.06 ± 0.010	0.13 ± 0.008	0.24 ± 0.051	0.08 ± 0.009
NaNO_3_	5	0.14 ± 0.009	**0.25** ± 0.010	0.08 ± 0.005	0.60 ± 0.013	**1.07** ± 0.015	0.42 ± 0.022
	25	0.09 ± 0.005	0.15 ± 0.026	0.05 ± 0.004	0.33 ± 0.015	0.61 ± 0.041	0.23 ± 0.018
	50	0.03 ± 0.008	0.05 ± 0.007	0.02 ± 0.002	0.20 ± 0.011	0.39 ± 0.028	0.15 ± 0.014
Urea	0	0.08 ± 0.005	**0.16** ± 0.012	0.05 ± 0.008	0.19 ± 0.010	0.35 ± 0.022	0.14 ± 0.009
	1	0.07 ± 0.006	0.13 ± 0.009	0.04 ± 0.005	0.37 ± 0.022	**0.73** ± 0.055	0.26 ± 0.019
	5	0.01 ± 0.001	0.04 ± 0.004	0.04 ± 0.007	0.05 ± 0.001	0.04 ± 0.001	0.03 ± 0.002

^*a*^ Data are the mean values of three independent biological replicates ± SD.

^*b*^ Bold numbers indicate the highest purity values obtained for phycobiliprotein pigments, with phycoerythrin showing the highest amount in all treatments.

### The effect of the oxidative stress (H_2_O_2_) on the growth and pigment profile

**Fig 9:**
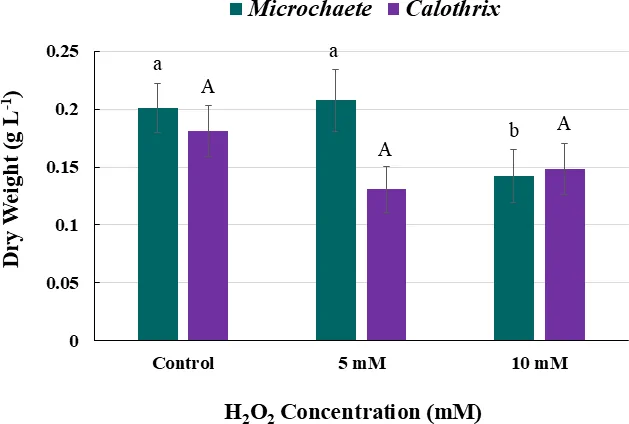
The effect of H_2_O_2_ on the dry weight of *Microchaete* and *Calothrix*. Data are mean values of three independent biological replicates. Different letters indicate significant differences (*P* ≤ 0.05).

Treatment with 5 mM H_2_O_2_ had a lesser impact on the growth of *Microchaete* compared to *Calothrix* (Fig. 9). The reduction in dry weight of *Calothrix* at concentrations of 5 and 10 mM H_2_O_2_ was not statistically significant compared to the control. However, exposure to 10 mM H_2_O_2_ significantly decreased the dry weight in *Microchaete*, resulting in biomass 1.4 times less than the control.

**Fig 10:**
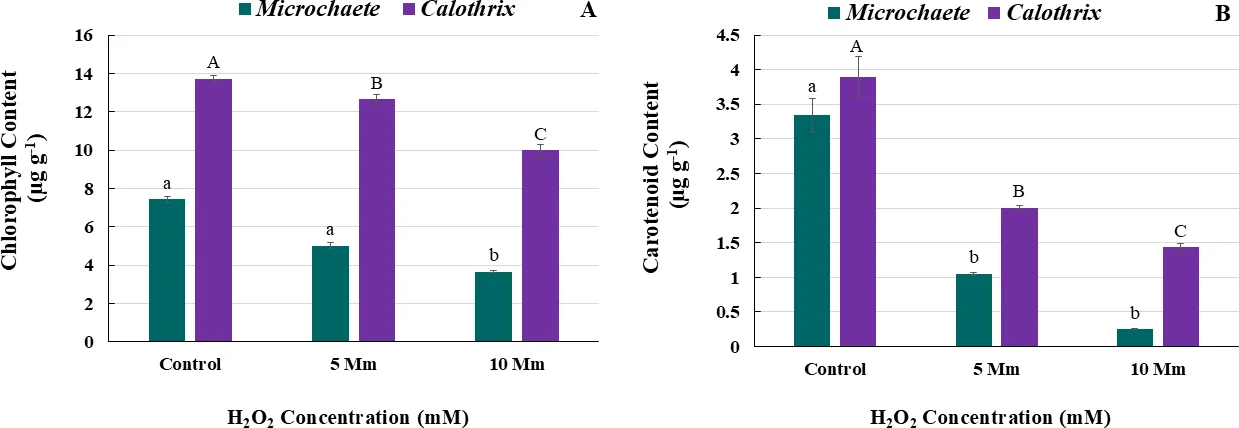
The effect of H_2_O_2_ on chlorophyll (**A**) and carotenoid (**B**) content in *Microchaete* and *Calothrix*. Data are mean values of three independent biological replicates. Different letters indicate significant differences (*P* ≤ 0.05).

H_₂_O_₂_ caused a significant decrease in chlorophyll and carotenoid content in *Microchaete* and *Calothrix* (Fig. 10A and B). However, the decrease in chlorophyll concentration at 5 mM H_₂_O_₂_ in *Microchaete* was not statistically significant compared to the control. In both strains, the reduction in carotenoid content was more pronounced than the decrease in chlorophyll content.

**Fig 11:**
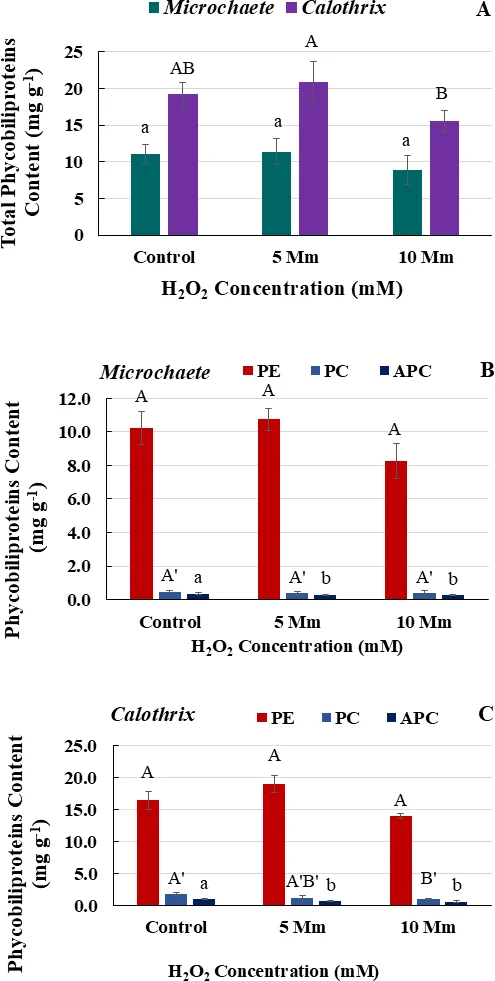
The effect of H_2_O_2_ on the content of total phycobiliproteins (**A**), phycocyanin, phycoerythrin, and allophycocyanin, in *Microchaete* (**B**) and *Calothrix* (**C**). Data are mean values of three independent biological replicates. Different letters indicate significant differences (*P* ≤ 0.05).

At concentrations of 5 and 10 mM H_₂_O_₂_, no significant effect on total PBP content was observed in *Microchaete* compared to the control (Fig. 11A). A similar trend was found for PE and PC levels in this cyanobacterium (Fig. 11B). *Calothrix* grown in the presence of 10 mM H_2_O_2_ showed a significant decrease in total PBP content compared to the 5 mM H_₂_O_₂_ treatment (Fig. 11A). However, PE content in *Calothrix* remained unaffected by H_₂_O_₂_ exposure (Fig. 11C). In contrast, H_2_O_2_ treatment resulted in a significant reduction in APC concentration in both cyanobacteria.

The highest content of total PBPs and PE in *Microchaete* (11.39 and 10.74 mg L^−1^, respectively) and *Calothrix* (20.94 and 19.04 mg L^−1^, respectively) was recorded in cultures supplemented with 5 mM H_2_O_2_.

**Fig 12:**
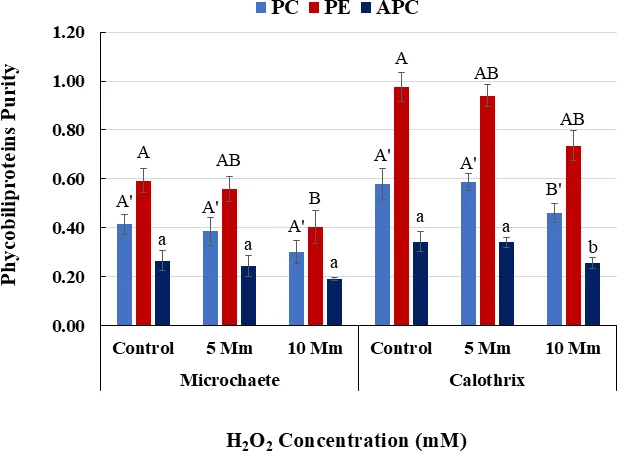
The effect of H_2_O_2_ on the purity of phycocyanin, phycoerythrin, and allophycocyanin in *Microchaete* and *Calothrix*. Data are mean values of three independent biological replicates. Different letters indicate significant differences (*P* ≤ 0.05).

At the lower concentration tested, H_2_O_2_ had no significant effect on the purity of PE, PC, and APC (Fig. 12). However, a significant decrease was observed at 10 mM, except for PC and APC in *Microchaete*.

## DISCUSSION

The production of biomass and photosynthetic pigments, particularly PBPs, in cyanobacteria is significantly influenced by environmental factors, such as light, salinity, and nutrient levels. Precise control of these variables is essential to enhancing cellular growth and metabolite accumulation for successful large-scale cultivation and biotechnological applications (30). This study on *Calothrix* and *Microchaete* revealed their specific responses to light, nitrogen availability, salinity, and oxidative stress.

Light conditions, such as intensity and photoperiod, are key factors affecting both biomass accumulation and pigment composition in cyanobacteria. Although higher light intensity often promotes growth, it may not be optimal for pigment production (31). Our results demonstrated that both *Microchaete* and *Calothrix* produced maximum biomass under high light intensity (100 µmol photons m^−2^ s^−1^) (Fig. 1). At the molecular level, light intensity regulates gene expression through photoreceptors such as cyanobacteriochromes and phytochromes, which sense changes in light quantity and quality and transmit signals to transcriptional regulators (e.g., RpaA/RpaB systems) that control photosystem components (psaA/B and psbA/D). Under high photon flux, upregulation of these photosystem genes enhances electron transport and ATP/NADPH production, enabling increased carbon fixation and biomass accumulation (18, 32). However, excessive light also generates ROS at Photosystem II, triggering redox-sensitive signaling that downregulates light-harvesting genes (including phycobiliprotein operons cpcBA and cpeBA) to mitigate photodamage (9). This molecular balancing act explains why high light can promote growth yet depress pigment synthesis in certain contexts.

This finding is consistent with studies on *Phormidium* sp. and *Cyanothece* sp., which also exhibited the highest yields under 160 μmol photons m^−2^ s^−1^ of white light compared to 40 μmol photons m^−2^ s^−1^ (30). Similarly, Niangoran et al. (31) reported increased biomass production of *Spirulina platensis* under constant illumination at 160 μmol m^−2^ s^−1^, relative to 80 μmol m^−2^ s^−1^. However, this contrasts with other cyanobacteria, such as *Nostoc* spp., which grew optimally under low-light conditions (12.5–25 µmol photons m^−2^ s^−1^) (33). These findings indicate the diversity of photo-adaptive strategies across the cyanobacterial phylum. The impact of light intensity on the biosynthesis and composition of pigments varies significantly across different cyanobacterial strains. Understanding this effect is important to elucidate the correlation between light exposure and cellular efficiency (34).

In our study, both examined cyanobacteria exhibited pigment modulation in response to changing light conditions. An increase in our light intensity led to a statistically non-significant reduction in chlorophyll and carotenoid content in *Microchaete*. In contrast, *Calothrix* showed significantly higher chlorophyll production under 50 µmol photons m^−2^ s^−1^, while its carotenoid levels remained unaffected by changes in light intensity (Fig. 2). Chlorophyll and carotenoid levels are controlled transcriptionally and post-transcriptionally by light and redox status. High intensities and resulting ROS activate protective pathways that inhibit enzymes in the chlorophyll biosynthesis pathway (e.g., chlL, chlB, and chlN) and shift metabolic flux toward carotenoid biosynthesis genes (crtB and crtP) as a photoprotective response (35, 36). In *Calothrix*, moderate light may optimize the balance between photoreceptor-mediated induction of chlorophyll biosynthesis and controlled ROS formation, thus sustaining chlorophyll levels. The relatively stable carotenoid content suggests post-translational regulation of carotenoid cyclases and desaturases, which ensures ROS scavenging without depleting precursor pools under fluctuating light.

Studies on different *Nostoc* species have demonstrated that reduced light levels promote greater synthesis of chlorophyll *a* and PBPs. When exposed to higher light intensity, the concentration of these pigments typically declined. Conversely, carotenoids generally accumulated under higher light conditions, emphasizing their function in protecting cyanobacteria from light-induced stress. In *Anabaena* sp., on the 15th day, chlorophyll *a*, total carotenoids, and PC content reached their peaks under low-light conditions, with significantly higher concentrations than those observed under high-light exposure (37). Niangoran et al. (31) reported more carotenoid and chlorophyll production in *S. platensis* grown under a constant light intensity of 80 μmol m^−2^ s^−1^ compared to 160 μmol m^−2^ s^−1^ of white light. A decrease in chlorophyll and carotenoid content with increasing light intensity in *Spirulina* has also been documented in other studies (38, 39). However, *S. platensis* showed higher production of PC under elevated light intensity (31).

As light intensity increases, chlorophyll content in cells generally declines, even as the growth rate accelerates. In contrast, under low-light conditions, cyanobacteria adapt by enhancing the production of light-harvesting pigments, including chlorophyll (40). Moreover, studies suggest that increased light intensity can reduce the synthesis of PBPs, especially PC (34). This inverse relationship between light intensity and total PBP content forms the basis of cyanobacterial photoacclimation (9). According to our results, the highest levels of total PBPs, PE, PC, and APC, in *Calothrix* and *Microchaete* were recorded under low (10 µmol photons m^−2^ s^−1^) and moderate (50 µmol photons m^−2^ s^−1^) irradiances, respectively (Fig. 3 and 4). This response reflects a well-established adaptive mechanism whereby cyanobacteria increase the size or number of light-harvesting antennae (phycobilisomes) to maximize photon capture and improve light absorption under low-light conditions. This strategy enables the maintenance of photosynthetic efficiency with minimal energy (30, 41). Phycobiliprotein biosynthesis is governed by tightly regulated gene clusters (e.g., cpcBA for PC, cpeBA for PE, and apcAB for APC), whose transcription is modulated by light intensity via two-component systems (RcaE/F) and chromatic acclimation regulators (42, 43). Under low light, increased expression of these operons leads to larger or more numerous phycobilisomes, maximizing photon capture. In contrast, high light activates ROS-activated transcriptional repressors that decrease PBP gene expression to avoid overexcitation and ROS accumulation at PSII (32).

At the post-transcriptional level, the activity of phycobilin lyases (cpcE/F and cpeS) affects the efficiency of chromophore attachment, influencing the purity and stability of PBPs observed in the pigments profiles.

Conversely, the observed decrease in PBP content under high-light intensity (100 µmol photons m⁻² s⁻¹) indicates a crucial photoprotective strategy. By reducing the absorption capacity, cells minimize the risk of photoinhibition and oxidative damage caused by excess energy uptake (30). This adaptive regulation of the photosynthetic apparatus, known as complementary chromatic adaptation, optimizes photosynthesis by maximizing the use of available light and is essential for survival in dynamically changing light environments (9). In *Nostoc* spp., low light intensities promote accumulation of light-absorbing pigments, including PBPs and chlorophyll *a*. As light availability increased from 10 to 150 μmol m^−2^ s^−1^, the concentrations of these pigments declined, likely as a protective response against damage induced by reactive oxygen species. However, the levels of carotenoid tended to rise when chlorophyll *a* and PBPs decreased, suggesting a compensatory mechanism for photoprotection (44). A study on *Limnospira fusiformis* revealed that illumination of 52 µmol photons m^−2^ s^−1^ promoted PC production, whereas a slight increase to 60 µmol photons m^−2^ s^−1^ resulted in a significant reduction in this metabolite (34).

While photoperiod did not significantly influence the growth of either *Microchaete* or *Calothrix* (Fig. 1B), it emerged as a critical factor in pigment metabolism (Fig. 2C and 3B). The different responses of the two cyanobacteria are noteworthy. *Microchaete* optimized the production of carotenoids under a long-day cycle (16:8 h light-dark), whereas the duration of light exposure had no significant effect on chlorophyll and PBP levels in this strain. In contrast, *Calothrix* exhibited its highest carotenoid and PBP production under a short-day cycle (8 h of light) (Fig. 4C, D, 3C, and D). Photoperiod impacts gene expression rhythms via the cyanobacterial circadian clock (KaiABC oscillator). Oscillations in KaiC phosphorylation state drive rhythmic expression of photosynthesis and pigment biosynthesis genes, aligning metabolic activity with the light-dark cycle (45).

Species-specific photoperiod sensitivity reflects differences in clock output pathways and light signaling strength, affecting the expression timing of carotenoid pathway genes (crtR) and PBPs operons (cpc and cpe) (46), thus generating the observed variation in pigment accumulation between *Microchaete* and *Calothrix* under different light regimes.

These results revealed that there is no universal optimal photoperiod; rather, photoperiod sensitivity is a species-specific trait. These findings collectively affirm that rhythmic light-dark cycles are a powerful tool for modulating light-harvesting capacity and metabolic pathways in cyanobacteria (47, 48). It has been reported that a 16:8 h light-dark cycle improved the growth, pigment production, protein levels, photosynthetic performance, and overall physiology in *Synechocystis* sp. PCC 6803, whereas continuous exposure to photosynthetically active radiation negatively affected photosynthetic pigment levels (47). In *Nostoc* sp., alternating exposure to light and dark led to enhanced growth and increased pigment composition (48). In *Phormidium*, elevated white light intensity promoted the accumulation of total carotenoids and β-carotene (30). The highest biomass and PC production in *S. platensis* were also achieved under continuous light (24:00 h) at an intensity of 160 μmol m^−2^ s^−1^, while the highest levels of chlorophyll and carotenoids were recorded under a 16:8 h cycle (31). Overall, an 8-h light period appears optimal for maximizing the concentration of PBPs in both *Calothrix* and *Microchaete*.

Abiotic stresses, such as salinity, are recognized as key triggers for enhanced production of valuable metabolites in cyanobacteria (49). It is well known that maintaining appropriate salt concentration is important for cellular function, ion regulation, membrane potential, osmotic balance, and overall metabolic activity. Therefore, identifying the optimal salinity range for maximizing biomass and the production of high-value biochemicals could enhance production potential and reduce cultivation costs (13). In the present study, NaCl supplementation enhanced biomass production in both cyanobacteria strains tested. *Microchaete* demonstrated greater salt tolerance than *Calothrix*, showing no growth inhibition up to 100 mM NaCl, whereas *Calothrix* exhibited a non-significant decrease in dry weight at concentrations above 50 mM NaCl. Additionally, the growth of *Microchaete* and *Calothrix* was stimulated by the addition of 25 mM NaNO_3_. Furthermore, 1 mM urea was optimal for the growth of *Calothrix*, while *Microchaete* grew best in a urea-free medium (Fig. 5). It has been reported that *Nostoc ellipsosporum* exhibits greater tolerance to NaCl and produces more biomass under saline conditions compared to *Nostoc piscinale*. Moderate NaCl concentrations were also found to significantly enhance PBP levels in both species (50). *Thermosynechococcus* sp. showed decreased biomass production with increasing NaCl concentrations (8, 18, and 29 g L^−1^) (51). An increase in biomass has been demonstrated in *Nostoc* sp. PCC 7423 when cultured in the presence of 5 mM NaNO_3_ and 2 g L^−1^ glucose (52). Studies on high-salinity-tolerant cyanobacteria, such as *Synechocystis* PCC 6803 and *Euhalothece* sp., emphasize the central role of salt homeostasis in sustaining metabolism (53, 54). Increased levels of Na^+^ and Cl^−^ in the culture medium disrupt cellular ion homeostasis, leading to osmotic stress that significantly impairs growth and biomass production (55). The reduction in cyanobacterial growth under increasing salinity is attributed not only to a decline in photosynthetic efficiency and inhibition of the electron transport chain but also to the metabolic redirection of energy toward active Na^+^ ion extrusion and the biosynthesis of carbohydrates as osmoprotectants. Disruption in ion homeostasis can adversely affect the photosystem II reaction center, leading to changes in the oxygen-evolving complex and triggering the generation of reactive oxygen species (55, 56).

Salt stress triggers osmoregulatory and ion homeostasis networks involving Na^+^/H^+^ antiporters (nhaS family), K^+^ transporters, and compatible solute biosynthesis (osmoprotectants such as glycine betaine and sucrose) to stabilize cellular macromolecules and membranes (15). At moderate NaCl, controlled ionic influx can signal via redox-sensitive regulators (e.g., Hik33) that coordinate the expression of stress-responsive pigment genes to maintain phycobilisome integrity. Excess salinity increases ROS and activates stress kinases that repress energy-intensive biosynthesis pathways, including PBPs, shifting metabolism toward maintenance and survival rather than pigment production (55).

Nitrogen is an essential nutrient for cyanobacteria, playing a crucial role in their growth rate and the organization of light-harvesting systems, especially PBPs. The optimal nitrogen source varies among cyanobacterial strains. Nitrate is commonly used in most cultivation protocols and is a key component of many standard culture media due to its availability and metabolic efficiency. It undergoes sequential reduction to nitrite and then to ammonium, which is ultimately incorporated into cellular storage compounds (52). Interestingly, *Arthrospira platensis* has been reported to prefer urea over nitrate as its nitrogen source. In our study, modifying NaNO_3_ concentrations had no significant effect on chlorophyll and carotenoid levels in *Calothrix*, whereas in *Microchaete*, peak carotenoid production was achieved at 25 mM NaNO_3_. Besides, 1 mM urea effectively increased chlorophyll and carotenoid production in both cyanobacteria. Salinity stress, including salinity induced by ionization of NaNO_3_, has been shown to influence pigment production, as higher salinity levels tend to promote carotenoid accumulation as a protective response against osmotic stress (Fig. 6).

Nitrogen assimilation is tightly linked to pigment biosynthesis via the global nitrogen regulator NtcA. NtcA controls the transcription of nitrate/nitrite transporters (nrtABCD), nitrate reductase (narB), and the phycobiliprotein gene clusters (cpc, cpe, and apc) ().

Under moderate nitrate/urea, balanced ammonium production supports the synthesis of tetrapyrrole precursors for chlorophyll and bilins for PBPs. Excess nitrogen, however, represses NtcA-dependent activation of these operons, favoring growth over pigment production and explaining the decline in PBPs at higher NaNO_3_ (52).

This is consistent with cyanobacterial adaptations to extreme environmental conditions, where carotenoids act as antioxidants to prevent cellular damage (46). Other studies have also reported increased production of protective carotenoid pigments under high-salinity conditions. Kosourov et al. (28) showed that carotenogenesis in *Calothrix* varies with the nitrogen status of the cultures. Specifically, echinenone accumulates during diazotrophic growth at the expense of β-carotene, while severe nitrogen deficiency combined with high CO_2_ supply promotes the accumulation of glycosylated and hydroxylated carotenoids. Increasing salinity from 0 to 0.2 M in BG11 medium resulted in elevated total carotenoid levels in *Synechocystis* sp. CCNM 2501 from 5.82 to 7.05 mg·g^−1^; however, further salinity elevation to 1 M led to a subsequent decline (57).

According to our results, exposure to 50 mM NaCl significantly increased PBP production in both *Calothrix* and *Microchaete*. The nitrogen source (NaNO_3_ or urea) also had a notable effect on pigment profiles. The highest PBP levels were observed at the lowest nitrate concentration (5 mM) in both strains (Fig. 7); however, this did not lead to high biomass production (Fig. 5A and B). Moreover, treatment with 1 mM urea markedly increased PBP content in *Calothrix*, whereas higher urea concentrations resulted in a significant reduction (Fig. 7C). It appears that 25 mM NaNO_3_ may redirect cellular energy toward growth, while PBP biosynthesis remains highly sensitive to elevated nitrate levels, showing a decline in both cyanobacteria. Consistent with these trends, 50 mM NaCl significantly increased the concentrations of PE, PC, and APC in both *Microchaete* and *Calothrix*. However, at 100 mM NaCl, both cyanobacteria exhibited a considerable decrease in PBP components compared to controls, indicating a threshold beyond which salinity stress impairs pigment synthesis (Fig. 8A and B). The content of PE, PC, and APC in both cyanobacteria declined progressively as the concentration of NaNO_3_ in the medium increased, with optimal pigment production observed at 5 mM NaNO_3_ (Fig. 8C and D). In *Microchaete*, 1 mM urea did not significantly affect PE, PC, or APC levels, whereas in *Calothrix*, this concentration significantly enhanced all three pigments. In contrast, treatment with 5 mM urea caused a pronounced decrease in these pigments in both strains (Fig. 8E and F). These observations are consistent with existing reports, highlighting the essential role of nitrogen in PBP biosynthesis. In the marine cyanobacterium *Synechocystis salina*, optimal biomass and PBP production, except for PE, were predicted at lower NaCl concentrations (10 g L⁻¹), while PE exhibited peak productivity at 25 g L^−1^ NaCl (55). Cyanobacterium *Fischerella* sp. produced the highest amount of PBPs using nitrite as a nitrogen source (58). Olvera-Ramírez (59) reported that the growth and biomass production in *Calothrix* were unaffected by initial nitrogen levels in the medium (0, 0.75, and 1.5 g/L), although reduced nitrate concentrations led to an increase in β-carotene content (59).

The highest growth rate, chlorophyll *a*, and PBP content in *A. platensis* were achieved in medium containing NaNO_3_ compared to KNO_3_ or NH_4_Cl (60). Lee et al. (49) investigated photosynthetic pigment production in *Synechocystis* sp. PCC 7338 under varying NaCl concentrations (0, 0.4, 0.8, and 1.2 M) and reported the highest content of chlorophyll *a*, APC, and PE at 1.2 M NaCl treatment. Cottas et al. (61) examined the effect of BG11^0^ medium supplemented with glucose and NaNO_3_ on PC production in *Anabaena variabilis*, identifying the highest yield with the combined application of 0.5 g L⁻¹ glucose and 2 mM NaNO_3_. In *Nostoc* sp. PCC 7423, peak PC content was achieved under supplementation with 5 mM NaNO_3_ and 2 g L^−1^ glucose (52). Simeunović et al. (62) reported that *Nostoc* and *Anabaena* strains cultivated in nitrogen-free media exhibited significant differences in PC and APC content compared to nitrogen-supplemented cultures, while PE and total PBP levels remained largely unchanged. It has been demonstrated that salinity stress can reduce PC concentration and thereby disrupt the energy transfer between PBPs and Photosystem II (63).

Our results showed that H_₂_O_₂_ exposure did not significantly affect biomass production in *Calothrix*, whereas the growth of *Microchaete* was markedly inhibited in the presence of 10 mM H_₂_O_₂_ (Fig. 9). The reduction in chlorophyll and carotenoid content in both *Calothrix* and *Microchaete* upon H_₂_O_₂_ treatment aligns with existing literature, which demonstrates that H_₂_O_2_, as a reactive oxygen species, induces oxidative stress that directly damages the photosynthetic system. Specifically, it can disrupt the electron transfer chain in Photosystem II and interfere with its recovery, making cells more susceptible to photoinhibition (64). Given that chlorophyll is the primary pigment responsible for light absorption during photosynthesis, its degradation is a direct consequence of this oxidative damage. Carotenoids, which have a protective role by scavenging ROS, are also susceptible to oxidative degradation (35). The observed reduction in their content suggests that the antioxidant systems of these cyanobacteria were insufficient to counteract the stress induced by H_₂_O_₂_, leading to cellular damage.

The pigment degradation, despite continued growth, implies a reallocation of energy, perhaps prioritizing survival over optimal photosynthetic performance. H_₂_O_₂_ and ROS cause oxidative modifications to proteins, lipids, and nucleic acids. In photosynthesis, ROS damage the D1 and D2 proteins of Photosystem II, impair electron transfer, and increase photoinhibition (35).

Cyanobacteria deploy antioxidant enzymes (catalase, superoxide dismutase, and peroxiredoxins) regulated by ROS-responsive transcription factors (e.g., PerR and OxyR) to detoxify ROS. At moderate ROS levels (5 mM H_₂_O_₂_), sublethal stress can induce hormetic upregulation of photoprotective pigments and PBPs via redox signaling, enhancing ROS scavenging capacity (64, 65).

At higher concentrations (≥10 mM), ROS exceed detoxification capacity, leading to pigment oxidation, phycobilisome protein degradation, and repression of pigment biosynthesis gene expression. Differential species sensitivity likely reflects variation in antioxidant enzyme capacity and regulatory circuit strength.

This adaptive response is commonly observed among prokaryotic phototrophs under oxidative stress conditions (35, 66). These findings are consistent with previous studies on other cyanobacteria. *Nostoc punctiforme* and *Anabaena* sp. exhibited similar declines in chlorophyll *a* following H_₂_O_₂_ exposure (65). In contrast, a study on the marine cyanobacterium *Synechococcus aeruginosus* reported a temporary increase in chlorophyll and carotenoid content during short exposure (15–30 min) to H_₂_O_2_, followed by a subsequent decline (67).

According to our results, treatment with 5 mM H_2_O_2_ led to a slight, though statistically insignificant, increase in total PBP and PE levels, whereas exposure to higher concentrations (10 mM) resulted in a decline. However, H_₂_O_₂_ caused a significant decrease in APC concentrations in both cyanobacteria. While the PC content in *Microchaete* remained unchanged, *Calothrix* exhibited a significant reduction in PC levels at 10 mM H_₂_O_₂_ compared to the control (Fig. 11). The results suggest that in both cyanobacteria, PBP production under oxidative stress is less sensitive than chlorophyll and carotenoid synthesis (Fig. 10). This points to a possible biphasic response to oxidative stress: at lower levels (5 mM), H_₂_O_₂_ may stimulate pigment synthesis as a protective mechanism, whereas higher concentrations result in cellular damage. PBPs, which play an important role in light energy harvesting, are highly regulated and responsive to various environmental stresses. The slight increase at lower H_₂_O_₂_ levels may reflect a hormesis effect, where low levels of stress stimulate an adaptive response (64). In cyanobacteria, such as *Nostoc*, PBPs also contribute to the antioxidant defense system, suggesting that their increased levels could be a temporary adaptive response to scavenge ROS and protect the photosynthetic apparatus. In contrast, the decrease in PBP content at elevated H_₂_O_₂_ levels indicates cellular damage. PBPs are embedded within phycobilisomes, which are highly susceptible to oxidative attack. Their degradation under intense stress conditions likely contributes to diminishing the overall efficiency of photosynthesis (35). Exposure to H_₂_O_₂_ has been shown to reduce growth and biomass in *A. platensis*, while concentrations up to 40 μM caused non-significant increases in chlorophyll *a*, PC, total PBPs, and carotenoid content (68). Studies on *N. punctiforme* and *Anabaena* sp. have revealed a clear difference in their tolerance to H_₂_O_₂_, with *Nostoc* exhibiting greater tolerance due to higher catalase activity (65). This finding demonstrates that cyanobacterial responses to H_₂_O_₂_ vary between species and are closely related to the effectiveness of their antioxidant systems and enzymatic detoxification pathways such as catalase and superoxide dismutase. Although limited data are available for *Calothrix*, reductions in Photosystem II efficiency under UV-B-induced oxidative stress suggest that its pigment systems may exhibit comparable sensitivity (69).

Overall, our findings indicate that H_₂_O_₂_ primarily targets photosynthetic pigments in *Calothrix* and *Microchaete* without significantly inhibiting growth, a pattern also demonstrated in related genera such as *Nostoc* and *Anabaena* (65). This reveals a complex physiological response, in which H_₂_O_₂_ affects cellular functions of cyanobacteria without a significant impact on their growth. It seems that a controlled application of oxidative priming could optimize PBP production while preserving cell viability.

In conclusion, this study elucidated the physiological responses of *Calothrix* and *Microchaete* to varying light intensities, photoperiods, nitrogen availability, salinity, and oxidative stress, offering useful insights for optimizing cultivation strategies. Both strains achieved maximal biomass under 100 µmol photons m⁻² s⁻¹, though pigment synthesis exhibited strain-specific sensitivity to light and nutrient conditions. *Calothrix* showed enhanced chlorophyll content at 50 µmol photons m⁻² s⁻¹ and optimal PBP production under low light and short photoperiods, while *Microchaete* favored moderate light and longer exposure. Nutrient supplementation with NaCl and NaNO_3_ enhanced biomass and pigment levels in both strains, whereas urea had divergent effects, stimulating *Microchaete* but inhibiting *Calothrix*. Oxidative stress reduced pigment content in both strains, with *Microchaete* exhibiting greater sensitivity. The differential pigment biosynthesis and biomass production observed between the two cyanobacteria underscore their unique adaptive capacities, with *Calothrix* favoring pigment accumulation under varied stress conditions.

## MATERIALS AND METHODS

*Microchaete* sp. strain ISC13 and *Calothrix* sp. strain ISC65 were obtained from the cyanobacteria collection of the Research Institute of Applied Sciences, ACECR, Tehran, Iran (70, 71).

All experiments were conducted under sterile conditions. Stock cultures were kept on sterile agar plates. Cultures were progressively scaled up from agar plates to 100 mL and then to 2 L over 2 months, with subculturing at 2-week intervals. Cyanobacterial cultures in the logarithmic growth phase were used as inocula. To prevent contamination during aeration, the air inlet line was equipped with sterile 0.22 μm filters.

For biomass production, cyanobacteria were cultured in BG11^0^ medium (BG11 medium without nitrate and containing 1.5 g L^−1^ NaCl) and maintained at 25°C ± 2°C under a white light intensity of 75 µmol photons m^−2^ s^−1^, with a 16:8 h light:dark photoperiod in continuous aeration for 15 days (72). In all experiments, biomass and the content of pigments, including PBPs, PE, PC, APC, chlorophyll, and carotenoids, were measured after 15 days of cultivation.

### The effect of light intensity and photoperiod on the growth and pigment profile

To investigate the effect of light intensity, *Microchaete* and *Calothrix* (2 g L^−1^ fresh weight) were cultured in BG11^0^ medium under a 16:8 h photoperiod. Cultures were exposed to white light at intensities of 10, 50, and 100 µmol photons m⁻² s⁻¹.

To examine the influence of different photoperiod regimes, cyanobacterial cultures at a concentration of 1.5 g L^−1^ (fresh weight) were grown under three light-dark cycles, 8:16, 16:8, and 24:0, each at a white light intensity of 50 µmol photons m^−2^ s^−1^.

### The effect of nutrients and salinity on the growth and pigment profile

*Microchaete* and *Calothrix* were cultured under varying nitrogen and salinity treatments in a medium containing NaCl (Na^+^), NaNO_3_ (NO_3_^−^), and urea, as shown in Table 2. The final Na^+^ concentration was calculated from NaCl and NaNO_3_ in the media. Cultures were maintained under a 16:8 light-dark cycle of white light (50 µmol photons m^−2^ s^−1^).

**TABLE 2:** Different nutrients and salinity treatments applied to the *Microchaete* and *Calothrix* culture medium

Treatments	Na^+^ (mM)	NO_3_^−^ (mM)	Urea (mM)
Control	0	0	0
1	25	0	0
2	25	0	1
3	25	0	5
4	50	5	0
5	50	25	0
6	50	50	0
7	100	0	0

### The effect of oxidative stress on the growth and pigment profile

Hydrogen peroxide at concentrations of 5 and 10 mM was used as an oxidant and introduced into the culture medium at the beginning of the first and second weeks of cultivation. Cyanobacteria were grown under white light at an intensity of 50 µmol photons m^−2^ s^−1^, following a 16:8 h light-dark cycle.

### Pigment content assay

Due to the heterogeneous and aggregated growth of cyanobacteria cells, pigment assays were performed on dry weight biomass instead of selecting a volumetric unit (mL). In this case, 1 mL of saline water (0.85% NaCl) for PBP extract and 1 mL of methanol for chlorophyll and carotenoid extract were added to 10 mg dried samples separately. Methanol samples were kept at 4°C overnight, and PBP samples were subjected to three cycles of freeze-thawing at −20°C and 4°C. All samples were centrifuged at 9,390 × *g* for 10 min, and the absorbance of supernatants was read at 461, 650, and 665 nm for methanolic extracts and at 280, 562, 615, 652, and 680 nm for PBPs by BioTek Epoch microplate spectrophotometer (14, 73). Pigments were calculated using the following equations:


$$\begin{array}{l} PC=\frac{{OD}_{615}-0.474\times {OD}_{652}}{5.34},\\ APC=\frac{{OD}_{652}-0.208\times {OD}_{615}}{5.09},\\ PE=\frac{{OD}_{562}-2.41[PC]-0.849[APC]}{9.62},\\ PBPs=PC+PE+APC,\\ Chlorophyll\;a=(16.5\times {OD}_{665})-(8.3\times {OD}_{650}),\\ Carotenoid=[{OD}_{461}-(0.046\times {OD}_{665})]\times 4. \end{array}$$

In addition, the purity of PBP pigments was estimated as follows:


$$\begin{array}{l} PE={OD}_{560}/{OD}_{280},\\ PC={OD}_{620}/{OD}_{280},\\ APC={OD}_{650}/{OD}_{280}. \end{array}$$

In these equations, OD refers to optical density in the mentioned wavelengths (14, 73).

### Statistical analysis

All experiments were conducted in triplicate using a completely randomized design. Data were analyzed with SPSS software, and mean values were compared using Duncan’s multiple range test at a 5% significance level.

## Supplementary Material

Reviewer comments

## Data Availability

The data sets used and/or analyzed during the current study are available from the corresponding author on reasonable request.

## SUPPLEMENTAL MATERIAL

OPEN PEER REVIEW reviewer-comments.pdf An accounting of the reviewer comments and feedback.

ASM does not own the copyrights to Supplemental Material that may be linked to, or accessed through, an article. The authors have granted ASM a non-exclusive, world-wide license to publish the Supplemental Material files. Please contact the corresponding author directly for reuse.
