# Supplementary material for: Changes in cyanobacterial growth and photosynthetic pigment under different conditions
Source: Microbiol Spectr. 2026 Jun 15;14(7):e03782-25. doi: 10.1128/spectrum.03782-25 (PMC13339967; doi:10.1128/spectrum.03782-25)
Supplement: Reviewer comments [file reviewer-comments.pdf]

AMERICAN SOCIETY FOR MICROBIOLOGY | Microbiology Spectrum

Changes in cyanobacterial growth and photosynthetic pigment under different conditions

Mohsen Seyedabadi, Arehzoo Zaker, Fatemeh Akhar, and Maryam Ameri

Corresponding Author(s): Maryam Ameri, Academic Center for Education Culture and Research

Review Timeline:

Submission Date:	November 22, 2025
Editorial Decision:	December 28, 2025
Revision Received:	April 11, 2026
Editorial Decision:	May 8, 2026
Revision Received:	May 11, 2026
Editorial Decision:	May 14, 2026
Revision Received:	May 14, 2026
Accepted:	May 19, 2026

Editor: Pedro Nuno Leão

Reviewer(s): Disclosure of reviewer identity is with reference to reviewer comments included in decision letter(s). The following individuals involved in review of your submission have agreed to reveal their identity: Dezhuang Gao (Reviewer #2)

Transaction Report:

(Note: With the exception of the correction of typographical or spelling errors that could be a source of ambiguity, letters and reports are not edited. The original formatting of letters and referee reports may not be reflected in this compilation.)

DOI: https://doi.org/10.1128/spectrum.03782-25

Re: Spectrum03782-25 (Changes in cyanobacterial growth and photosynthetic pigment under different conditions)

Dear Dr. Maryam Ameri:

Thank you for the privilege of reviewing your work. Below you will find my comments, instructions from the Spectrum editorial office, and the reviewer comments.

You will see that your manuscript requires substantial revisions before being further considered for publication. The reviewers raise important points regarding the experimental approach with which I agree. The reviewers were particularly emphatic regarding the presentation of results, which should be clearer and more robust.

Please return the manuscript within 60 days; if you cannot complete the modification within this time period, please contact me. If you do not wish to modify the manuscript and prefer to submit it to another journal, notify me immediately so that the manuscript may be formally withdrawn from consideration by Spectrum.

Revision Guidelines
To submit your modified manuscript, log into the submission site at https://spectrum.msubmit.net/cgi-bin/main.plex. Go to Author Tasks and click the appropriate manuscript title to begin. The information you entered when you first submitted the paper will be displayed; update this as necessary. Note the following requirements:

• Upload point-by-point responses to the issues raised by the reviewers in a file named "Response to Reviewers," NOT in your cover letter.
• Upload a compare copy of the manuscript (without figures) as a "Marked-Up Manuscript" file.
• Upload a clean .DOC/.DOCX version of the revised manuscript and remove the previous version.
• Each figure must be uploaded as a separate, editable, high-resolution file (TIFF or EPS preferred), and any multipanel figures must be assembled into one file.
• Any supplemental material intended for posting by ASM should be uploaded with their legends separate from the main manuscript. You can combine all supplemental material into one file (preferred) or split it into a maximum of 10 files with all associated legends included.

For complete guidelines on revision requirements, see our Submission and Review Process webpage. Submission of a paper that does not conform to guidelines may delay acceptance of your manuscript.

Data availability: ASM policy requires that data be available to the public upon online posting of the article, so please verify all links to sequence records, if present, and make sure that each number retrieves the full record of the data. If a new accession number is not linked or a link is broken, provide Spectrum production staff with the correct URL for the record. If the accession numbers for new data are not publicly accessible before the expected online posting of the article, publication may be delayed; please contact production staff (Spectrum@asmusa.org) immediately with the expected release date.

Publication Fees: For information on publication fees and which article types are subject to charges, visit our website. If your manuscript is accepted for publication and any fees apply, you will be contacted separately about payment during the production process; please follow the instructions in that e-mail. Arrangements for payment must be made before your article is published.

ASM Membership: Corresponding authors may join or renew ASM membership to obtain discounts on publication fees. Need to upgrade your membership level? Please contact Customer Service at Service@asmusa.org.

The ASM Journals program strives for constant improvement in our submission and publication process. Please tell us how we can improve your experience by taking this quick Author Survey.

Thank you for submitting your paper to Spectrum.

Sincerely,
Pedro Nuno Leão
Editor
Microbiology Spectrum

Reviewer #2 (Comments for the Author):

This paper provides important insights into the metabolic flexibility of two cyanobacteria by investigating the effects of key environmental factors-light, salinity, nitrogen source, and induced oxidative stress-on the composition of photosynthetic

pigments and biomass. It reveals the specific responses of Calothrix and Microchaete to light, nitrogen availability, salinity, and oxidative stress.

Some minor suggestions:

1. Why were Calothrix and Microchaete chosen as the study subjects, and not other cyanobacteria such as Microcystis?
2. Were the experiments conducted under sterile conditions? As far as I know, some bacteria might symbiotically interact with cyanobacteria or compete with them, and such interspecies relationships could be influenced by environmental factors.
3. I recommend the authors combine multiple bar charts together. Additionally, these bar charts do not show error bars.
4. Some experimental results do not show significant correlations, which raises some concerns, such as in lines 129 and 138.
5. Figures 1a and 1b only show the effects of three light intensities (10, 50, and 100) on cyanobacterial growth, which do not strongly support the statement that "the photoperiod did not significantly affect the growth of either cyanobacterium." Similarly, Figure 3b with only three light intensities does not provide sufficient evidence to support the claim that "light intensity did not significantly affect the purity of PE, PC, and APC in Microchaete."
6. The experimental results are mainly presented from a macroscopic perspective. I suggest the authors further validate and interpret the findings from a molecular and genetic standpoint.

Summary

This manuscript describes environmental and growth medium components on the maximization of biomass and resulting extracted potential natural products of the cyanobacteria *Microchaete* sp. strain ISC13, and *Calothrix* sp. strain ISC65 isolated from originally isolated from highly contaminated oil polluted soils. The authors explored key growth components (light, light intensity, nitrogen source), environmental factors like salinity which could promote or reduce biomass, and testing if oxidative stressors could aid in the production of valuable biomolecules. They also give insight into physiological adaptations these two strains have toward photoacclimation and stress tolerances in cyanobacteria.

Overview

This paper is the product of many separate experiments, in multiple pieces to understand which variables could be modified/adjusted for mass production or optimization.

They tested:

- light intensity and photoperiod
- nutrients and salinity
- oxidative stress

The writing in the paper at times was confusing (the Results and Discussion sections) with each strain sometimes having opposition reactions to each single experiment and discussed in that sequence. There were times, I was waiting to see if there were interactions experiments between the variables they tested. It seems like the paper was seeking to identify only main effects and not interactions. These single tests give indications toward developing a suitable starting point, but the paper described optimizing growing conditions.

Lights X Nutrients could interaction, that high light without sufficient nitrogen could cause photoinhibition and pigment degradation? But I don't think that was tested.

If these strains were isolated from freshwater or brackish in origin increasing salinity could also cause oxidative stress.

Major

These two cyanobacteria are strains of undescribed species of Calothrix and Microchaete. Cyanobacteria taxonomy is polyphasic and can be challenging. I think don't always think all species or strains with a genus is always representative.

Also I wonder under some of large amounts of nitrogen added, there might not have been carbon limitation (bicarbonate) in the medium that could cause a stoichiometric imbalance –

leading to stress? I don't think based on the methods, that bicarbonate was modified. But the C:N:P ratio of the culture I think would be highly skewed.

I can't tell at what scale were these grown in the methods? Are these large batch or smaller batch cultures? I feel the methods are missing several key details.

Minor

Literature cited in text: sometimes the authors are alphabetical, sometimes by date, sometimes newest to older.

Lines:

Line 98: Statement about were Microchaete and Calothrix are found widely, should be cited from some references.

Line 102: This strain holds… I think this could be better addressed as "Calothrix sp. 1SC65 holds considerable " instead of strain.

Line 105: beginning with *Microchaete,* is there a citation to back up that statement? Also is that true of all Microchaete species that have been studied?

Line 131: the line ends with *0.31 g L-1*. What unit of measure is this? Dry weight or fresh weight

Line 280: starting with *In contrasts, Calothrix showed higher chlorophyll production* … This follows a statement about non-significant reductions in Microchaete. So was this significant? (thinking in contrast)

Line 352: It has been reported that N. ellipsosporum. Needs the genus name spelled out at least the first its used in the text and can be abbreviated after. So should say Nostoc ellipsosporum.

Line 354: N. piscinale should be Nostoc piscinale. Same reason as above.

Lines 376 – 382. This paragraph was very confusing. I read it several times and I still I'm not sure quite what it is saying. Its starts off about nitrogen, and then suddenly salinity. Also nitrate promoted chlorophyll and carotenoid, but then the next sentence says it did not in Calothrix?

Line 497: I think these need to be listed as Microchaete sp. strain ISC13, and Calothrix sp. strain ISC65. Because strains can be very different in cyanobacteria and until genomes exist of these specific cultures their taxonomy could be dubious.

Lines 523: How were pigments analyzed, on a spectrophotometer, what model or make? What methods were used needs to added.

Lines 529-540: Where are these equations from? Why did you pick those wavelengths?

Dear editor,

We are very glad to have been given the opportunity to revise our manuscript. We carefully considered your comments as well as those offered by the reviewers. We want to extend our appreciation for taking the time and effort necessary to provide such insightful guidance.

We hope that these revisions improve the paper such that you and the reviewers now deem it worthy of publication in *Microbiology Spectrum*. Next, we offer detailed responses to your comments as well as those of the reviewers.

We look forward to your positive response.

Sincerely,

Maryam Ameri

Thank you very much for all your comments. We have modified the manuscript accordingly your suggestions, and detailed corrections are listed below point by point. Changes made in the manuscript are highlighted in yellow.

In-house editorial comment:

We tried to change the text in the same way as the respected editor asked.

Reviewer 1:

…It seems like the paper was seeking to identify only main effects and not interactions. These single tests give indications toward developing a suitable starting point, but the paper described optimizing growing conditions. Lights X Nutrients could interaction, that high light without sufficient nitrogen could cause photoinhibition and pigment degradation? But I don't think that was tested. If these strains were isolated from freshwater or brackish in origin increasing salinity could also cause oxidative stress.

Thank you for your valuable feedback. The necessary revisions have been implemented to the best of our ability. Regarding your comments, it is important to note that these cyanobacteria possess the ability to fix atmospheric nitrogen and could therefore be considered potential biofertilizers. Unfortunately, the use of chemical fertilizers in fields such as rice paddies can negatively impact the growth of these cyanobacteria. As shown in Table 1, the nutrition section examines the effect of different nutrient levels on the growth and metabolism of the two cyanobacteria strains. Furthermore, each section of the table includes a common control experiment that allows comparison and potential inference of interactions between the other treatments (highlighted in blue and red). Experiment #1 in the nutrition section serves as the control for the light treatments, under standard photic cultivation conditions (16:8 photoperiod and 50 μmol m^{-2} s^{-1} light intensity).

Unfortunately, the interaction experiment you suggested (e.g., light × nutrients) was not performed in the current study. However, it is a compelling suggestion for future research. As you know, this article contains a large volume of data that we are obligated to present fully.

Urea effect				Na effect				NO3 effect			
No.	Na	NO3	Urea	No.	Na	NO3	Urea	No.	Na	NO3	Urea
				Cont.	0	0	0				
**1**	**25**	**0**	**0**	**1**	**25**	**0**	**0**	**4**	**50**	**5**	**0**
2	25	0	1	**4**	**50**	**5**	**0**	5	50	25	0
3	25	0	5	7	100	0	0	6	50	50	0

As you correctly pointed out, certain conditions tested in our experiments can induce oxidative stress in cyanobacteria. Therefore, the addition of hydrogen peroxide could, to some extent, help predict their tolerance thresholds and physiological responses.

Major: These two cyanobacteria are strains of undescribed species of *Calothrix* and *Microchaete* . Cyanobacteria taxonomy is polyphasic and can be challenging. I think don't always think all species or strains with a genus is always representative. Also I wonder under some of large amounts of nitrogen added, there might not have been carbon limitation (bicarbonate) in the medium that could cause a stoichiometric imbalance – leading to stress? I don't think based on the methods, that bicarbonate was modified. But the C:N:P ratio of

the culture I think would be highly skewed. I can't tell at what scale were these grown in the methods? Are these large batch or smaller batch cultures? I feel the methods are missing several key details. Minor Literature cited in text: sometimes the authors are alphabetical, sometimes by date, sometimes newest to olde

Thank you for your insightful and detailed comments. We appreciate the opportunity to clarify several important aspects of our study.

Regarding the genus explanation:

Thanks for your comment. As mentioned in method section, *Microchaete* sp. strain ISC13 and *Calothrix* sp. strain ISC65 were obtained from the cyanobacteria collection of the Research Institute of Applied Sciences, ACECR, Tehran, Iran. Some studies related to mentioned cyanobacteria are added in the text like Soltani et al., 2012 and Amirlatifi et al., 2018. If it is necessary, I can add more related studies about these cyanobacteria however unrelated to nutritional treatment like the present study. We used cyanobacteria biomass in log phase for our experiments to conduct them in same way. I agree that some responses are species specific but it is not possible for us to identify the species of these genus, unfortunately.

Regarding the rationale for nitrogen source experimentation:

As the reviewer correctly notes, cyanobacteria play a crucial role in biological nitrogen fixation and contribute significantly to nitrogen fertilization in agricultural ecosystems such as rice paddies. However, these same agricultural lands are often simultaneously subjected to chemical nitrogen fertilization (both nitrate and ammonium sources). This dual input-biological nitrogen fixation alongside chemical fertilization-creates complex nitrogen dynamics in the field.

Therefore, the primary objective of our study was to investigate the growth responses of these nitrogen-fixing cyanobacteria under varying concentrations of different nitrogen sources (nitrate and ammonium). Understanding how these cyanobacteria behave in the presence of combined nitrogen sources is ecologically relevant and agriculturally important, as it reflects real-world conditions where fixed nitrogen from fertilizers coexists with biological nitrogen fixation.

Regarding carbon limitation and stoichiometry:

We acknowledge the reviewer's concern about potential carbon limitation and C:N:P stoichiometric imbalance. In our experimental setup, aeration of the cultures served as the primary source of carbon dioxide (CO_2) supply, and no additional carbon source (such as bicarbonate) was supplemented to the medium.

Regarding salinity control:

We would also like to clarify that an additional focus of our study was maintaining controlled salinity conditions by regulating sodium concentrations in the culture media. This was particularly

important given the nitrogen sources used (sodium nitrate and ammonium chloride) and their potential impact on osmotic balance.

Regarding culture scale and experimental details:

Thank you for highlighting the need for more detailed information about the culture conditions. In response to your comment, we have now added comprehensive details regarding culture volumes and experimental scale to the Methods section.

Specifically, we have clarified that:

- **Seed cultures** were initially grown in 100 mL volumes under sterile conditions.
- **Scale-up** was progressively performed from 100 mL to 2 L over a two-month period, with subculturing at two-week intervals.
- **Experimental cultures** were conducted in 1 L culture vessels containing sterile medium, inoculated with cyanobacterial cultures in the logarithmic growth phase.
- **Aeration** was provided through filtered air inlet lines (0.22 µm sterile filters) to maintain sterility while ensuring adequate CO_2 supply.

These details have been added to the revised manuscript to provide readers with a clear understanding of the experimental scale and conditions. We thank the reviewer for helping us improve the clarity and reproducibility of our methods.

Regarding Literature citation:

It is performed.

Line 98: Statement about were *Microchaete* and *Calothrix* are found widely, should be cited from some references.

It was performed. The reference was added.

Line 102: This strain holds… I think this could be better addressed as "*Calothrix* sp. 1SC65 holds considerable " instead of strain.

It was performed.

Line 105: beginning with *Microchaete* , is there a citation to back up that statement? Also is that true of all *Microchaete* species that have been studied?

Thanks for your comment. It was edited to *Microchaete* sp. CCU342 as Hemlata et al., 2014 reported at their study.

Line 131: the line ends with 0.31 g L-1. What unit of measure is this? Dry weight or fresh weight

Data is related to dry weight, and it was referred in the text.

Line 280: starting with In contrasts, *Calothrix* showed higher chlorophyll production … This follows a statement about non-significant reductions in *Microchaete* . So was this significant? (thinking in contrast)

Many thanks for your attention. It was state that ". In contrast, *Calothrix* showed significantly…"

Line 352: It has been reported that N. ellipsosporum. Needs the genus name spelled out at least the first its used in the text and can be abbreviated after. So should say *Nostoc ellipsosporum*.

As you commented, it was edited.

Line 354: N. piscinale should be *Nostoc piscinale*. Same reason as above.

It was performed.

Lines 376 – 382. This paragraph was very confusing. I read it several times and I still I'm not sure quite what it is saying. Its starts off about nitrogen, and then suddenly salinity. Also nitrate promoted chlorophyll and carotenoid, but then the next sentence says it did not in *Calothrix* ?

This paragraph was rewritten. As you know, $NaNO_3$ is an agent for salinity and nitrate alternation in media. Salinity stress, including salinity-induced by ionization of $NaNO_3$ was our mean in this part.

Line 497: I think these need to be listed as *Microchaete* sp. strain ISC13, and *Calothrix* sp. strain ISC65. Because strains can be very different in cyanobacteria and until genomes exist of these specific cultures their taxonomy could be dubious.

It was performed.

Lines 523: How were pigments analyzed, on a spectrophotometer, what model or make? What methods were used needs to added.

Thanks for your comment. Details were added in the method section. We used BioTek Epoch microplate spectrophotometer for our pigment absorbence.

Lines 529-540: Where are these equations from? Why did you pick those wavelengths?

The references were added.

Reviewer 2:

1. Why were *Calothrix* and *Microchaete* chosen as the study subjects, and not other cyanobacteria such as Microcystis?

Thank you for this important question. Our selection of *Calothrix* and *Microchaete* as the study subjects was based on several scientific considerations:

First, our research group has previously worked on *Spirulina* (one of the most well-known cyanobacteria), which provided us with a strong foundation in cyanobacteriology. Following the isolation of the cyanobacteria investigated in this study, we observed an interesting and diverse pigment profile in these two genera that warranted further investigation.

Second, and more significantly, both *Calothrix* and *Microchaete are nitrogen-fixing cyanobacteria*. Their ability to fix atmospheric nitrogen made them particularly attractive for our research, as we are interested in their potential application as biofertilizers in sustainable agriculture. This practical application guided our focus toward evaluating key parameters for pigment production and biomass accumulation.

Regarding the reviewer's mention of *Microcystis*, we acknowledge this genus as an important subject in phycology. However, *Microcystis* is well-documented as a toxin-producing cyanobacterium frequently associated with harmful algal blooms. Since our ultimate research goals involve potential food or agricultural applications, toxin-producing genera fall outside the scope of our current study objectives.

2. Were the experiments conducted under sterile conditions? As far as I know, some bacteria might symbiotically interact with cyanobacteria or compete with them, and such interspecies relationships could be influenced by environmental factors.

Thank you for raising this important point regarding sterility and potential microbial interactions. To address your concern: Yes, all experiments were conducted under strictly sterile conditions throughout the study. Our sterility maintenance protocol included the following steps:

1. **Initial culture maintenance:** Following the identification of the cyanobacterial strains, all stock cultures were maintained on sterile agar slants under controlled conditions.

2. **Inoculation procedure:** All transfers from solid to liquid media were performed under strictly sterile conditions inside a laminar flow hood to prevent contamination.

3. **Scale-up process:** The cultures were progressively scaled up from 100 mL to 2 L over a period of two months, with subculturing performed at two-week intervals. This gradual scale-up ensured culture purity at each stage.

4. **Experimental inoculation:** Only cyanobacterial cultures in the logarithmic growth phase were used as inocula for the experiments. One-liter culture vessels containing sterile medium were inoculated under the laminar flow hood.

5. **Aeration system:** To maintain sterility during aeration, the main air inlet line was equipped with sterile filters (0.22 µm) to prevent contaminants from entering the cultures while allowing adequate gas exchange.

These stringent sterility measures ensured that the observed growth and pigment production patterns resulted solely from the cyanobacterial strains themselves, without interference from contaminating bacteria that might otherwise compete or interact symbiotically.

We have now added a detailed description of our sterility protocols to the Methods section to clarify this important methodological aspect for readers.

3. I recommend the authors combine multiple bar charts together. Additionally, these bar charts do not show error bars.

Thank you for your valuable suggestion to improve the presentation of our data. In response to your recommendation:

1. Combining bar charts: We have carefully reviewed all figures and, where scientifically appropriate, combined multiple bar charts into single, more comprehensive figures to facilitate comparison and improve clarity. The revised figures now present related data together while maintaining visual clarity.

2. Error bars: We have added standard deviation bars to all bar charts throughout the manuscript, as originally requested. These error bars represent the variability observed across replicates and provide a clearer indication of data reliability and statistical significance.

All revised figures have been uploaded as separate, high-resolution files in accordance with the journal's formatting requirements. We believe these modifications have substantially improved the visual presentation and scientific rigor of our data. We thank the reviewer for this constructive feedback.

4. Some experimental results do not show significant correlations, which raises some concerns, such as in lines 129 and 138.

We found that higher light intensity increased biomass while reduced photosynthetic pigments include chlorophyll, carotenoid and phycobiliproteins. It was interesting for us, and we guess some exopolysaccharides (EPS) could be secreted from the cells. As we observed, these cyanobacteria

have heterogeneous and aggregated growth in their cells. Also, they are able to produce EPS. Unfortunately, we didn't measure EPS in our study but in another work of mine about the effect of Aluminum on *Scenedesmus* microalgae, Al induced oxidative stress while more biomass was achieved. Microscopic observation demonstrated aggregated cells (Ameri et al., 2020). Literature declares secretions like malate, oxalate, and citrates. So, perhaps some other metabolites can be produced and increase the biomass weight.

(Ameri, M., Baron-Sola, A., Khavari-Nejad, R. A., Soltani, N., Najafi, F., Bagheri, A., ... & Hernández, L. E. (2020). Aluminium triggers oxidative stress and antioxidant response in the microalgae Scenedesmus sp. *Journal of plant physiology*, *246*, 153114.

Ameri, M., Khavari-Nejad, R. A., Soltani, N., Najafi, F., Bagheri, A., & Seyedabadi, M. (2023). Characterization of the aluminum-resistant microalgae by screening industrial wastewater microorganisms.)

5. Figures 1a and 1b only show the effects of three light intensities (10, 50, and 100) on cyanobacterial growth, which do not strongly support the statement that "the photoperiod did not significantly affect the growth of either cyanobacterium." Similarly, Figure 3b with only three light intensities does not provide sufficient evidence to support the claim that "light intensity did not significantly affect the purity of PE, PC, and APC in *Microchaete* ."

Figures 1a and 1b show the effect of light intensities and **photoperiod** on biomass production separately. As figure 1b shows, photoperiod didn't affect biomass production significantly for both cyanobacteria.

Also, I didn't get your mean about figure 3b. it was observed that the purity of PE, PC, and APC in *Microchaete* was not affected by light intensity at **10, 50, and 100** µmol photons $m^{-2} s^{-1}$. Do you mean these intensities is not enough for our declaration or we should mention "in these light intensities"?

6. The experimental results are mainly presented from a macroscopic perspective. I suggest the authors further validate and interpret the findings from a molecular and genetic standpoint.

Thank you for this insightful comment regarding the molecular and genetic aspects of our study. We fully agree that investigating the molecular mechanisms underlying the observed phenotypic differences would provide valuable depth to this research. In response to your suggestion:

1. **Literature integration:** Following the reviewer's recommendation, we have expanded the Discussion section to incorporate relevant molecular and genetic studies from the existing literature. We now discuss potential genetic pathways and regulatory mechanisms that may

explain the observed differences in pigment production and biomass accumulation between *Calothrix* and *Microchaete*, drawing upon previously published genomic and transcriptomic data in related cyanobacteria.

2. **Study limitations:** We acknowledge that the current study is primarily phenotypic and physiological in nature, and we do not have molecular data (such as gene expression analysis or genomic characterization) to directly support mechanistic interpretations.

We believe these additions appropriately address the reviewer's concern while maintaining scientific honesty about the scope of the current study. The revised Discussion section now provides a more balanced perspective that connects our macroscopic observations with the broader molecular understanding of cyanobacterial physiology.

Re: Spectrum03782-25R1 (Changes in cyanobacterial growth and photosynthetic pigment under different conditions)

Dear Dr. Maryam Ameri:

Thank you for the privilege of reviewing your work. Below you will find my comments, instructions from the Spectrum editorial office, and the reviewer comments.

Please take into account Reviewer #2's suggestion, in response to your comment in the point-by-point response. Include "at these light intensities" in the proof stage.

Please return the manuscript within 60 days; if you cannot complete the modification within this time period, please contact me. If you do not wish to modify the manuscript and prefer to submit it to another journal, notify me immediately so that the manuscript may be formally withdrawn from consideration by Spectrum.

Revision Guidelines
To submit your modified manuscript, log into the submission site at https://spectrum.msubmit.net/cgi-bin/main.plex. Go to Author Tasks and click the appropriate manuscript title to begin. The information you entered when you first submitted the paper will be displayed; update this as necessary. Note the following requirements:

• Upload point-by-point responses to the issues raised by the reviewers in a file named "Response to Reviewers," NOT in your cover letter.
• Upload a compare copy of the manuscript (without figures) as a "Marked-Up Manuscript" file.
• Upload a clean .DOC/.DOCX version of the revised manuscript and remove the previous version.
• Each figure must be uploaded as a separate, editable, high-resolution file (TIFF or EPS preferred), and any multipanel figures must be assembled into one file.
• Any supplemental material intended for posting by ASM should be uploaded with their legends separate from the main manuscript. You can combine all supplemental material into one file (preferred) or split it into a maximum of 10 files with all associated legends included.

For complete guidelines on revision requirements, see our Submission and Review Process webpage. Submission of a paper that does not conform to guidelines may delay acceptance of your manuscript.

Data availability: ASM policy requires that data be available to the public upon online posting of the article, so please verify all links to sequence records, if present, and make sure that each number retrieves the full record of the data. If a new accession number is not linked or a link is broken, provide Spectrum production staff with the correct URL for the record. If the accession numbers for new data are not publicly accessible before the expected online posting of the article, publication may be delayed; please contact production staff (Spectrum@asmusa.org) immediately with the expected release date.

Publication Fees: For information on publication fees and which article types are subject to charges, visit our website. If your manuscript is accepted for publication and any fees apply, you will be contacted separately about payment during the production process; please follow the instructions in that e-mail. Arrangements for payment must be made before your article is published.

ASM Membership: Corresponding authors may join or renew ASM membership to obtain discounts on publication fees. Need to upgrade your membership level? Please contact Customer Service at Service@asmusa.org.

The ASM Journals program strives for constant improvement in our submission and publication process. Please tell us how we can improve your experience by taking this quick Author Survey.

Thank you for submitting your paper to Spectrum.

Sincerely,
Pedro Nuno Leão
Editor
Microbiology Spectrum

Reviewer #2 (Comments for the Author):

For suggestion 5, I suggest that the author use "at these light intensities" to make the statement more accurate.

Dear Editor,

I would like to sincerely thank you and the reviewers for your valuable comments and careful evaluation of my manuscript for possible publication in *Microbiology Spectrum*.

I have now revised the manuscript based on the reviewer #2 suggestion. All changes made in response to the reviewer's comments have been highlighted in blue for your convenience.

Additionally, after submitting the previous version, you asked me in a later email to format the references in a SPEC style. I had already done this within the main body of the text, but unfortunately, I did not apply this change to the reference list at the end of the manuscript. Please be assured that the references have now been formatted exactly according to your requested style throughout the entire manuscript.

I kindly ask you to consider the revised version for further processing.

Thank you once again for your time and understanding.

Sincerely,

Maryam Ameri

Reviewer 2:

Thank you for your precise comment. We have addressed your suggestion in the revised manuscript, with the change highlighted in blue.

Re: Spectrum03782-25R2 (Changes in cyanobacterial growth and photosynthetic pigment under different conditions)

Dear Dr. Maryam Ameri:

It appears that the correct files were not uploaded (also as indicated by you via email on the 11th of May). Please re-upload the correct files as soon as possible.

Revision Guidelines
To submit your modified manuscript, log into the submission site at https://spectrum.msubmit.net/cgi-bin/main.plex. Go to Author Tasks and click the appropriate manuscript title to begin. The information you entered when you first submitted the paper will be displayed; update this as necessary. Note the following requirements:

• Upload point-by-point responses to the issues raised by the reviewers in a file named "Response to Reviewers," NOT in your cover letter.
• Upload a compare copy of the manuscript (without figures) as a "Marked-Up Manuscript" file.
• Upload a clean .DOC/.DOCX version of the revised manuscript and remove the previous version.
• Each figure must be uploaded as a separate, editable, high-resolution file (TIFF or EPS preferred), and any multipanel figures must be assembled into one file.
• Any supplemental material intended for posting by ASM should be uploaded with their legends separate from the main manuscript. You can combine all supplemental material into one file (preferred) or split it into a maximum of 10 files with all associated legends included.

For complete guidelines on revision requirements, see our Submission and Review Process webpage. Submission of a paper that does not conform to guidelines may delay acceptance of your manuscript.

Data availability: ASM policy requires that data be available to the public upon online posting of the article, so please verify all links to sequence records, if present, and make sure that each number retrieves the full record of the data. If a new accession number is not linked or a link is broken, provide Spectrum production staff with the correct URL for the record. If the accession numbers for new data are not publicly accessible before the expected online posting of the article, publication may be delayed; please contact production staff (Spectrum@asmusa.org) immediately with the expected release date.

Publication Fees: For information on publication fees and which article types are subject to charges, visit our website. If your manuscript is accepted for publication and any fees apply, you will be contacted separately about payment during the production process; please follow the instructions in that e-mail. Arrangements for payment must be made before your article is published.

ASM Membership: Corresponding authors may join or renew ASM membership to obtain discounts on publication fees. Need to upgrade your membership level? Please contact Customer Service at Service@asmusa.org.

The ASM Journals program strives for constant improvement in our submission and publication process. Please tell us how we can improve your experience by taking this quick Author Survey.

Thank you for submitting your paper to Spectrum.

Sincerely,
Pedro Nuno Leão
Editor
Microbiology Spectrum

Dear Editor,

I would like to sincerely thank you and the reviewers for your valuable comments and careful evaluation of my manuscript for possible publication in *Microbiology Spectrum*.

I have now revised the manuscript based on the reviewer #2 suggestion. All changes made in response to the reviewer's comments have been highlighted in gray for your convenience. The tracked changes for these sections are also visible in the marked-up manuscript.

Thank you once again for your time and understanding.

Sincerely,

Maryam Ameri

Reviewer 2:

1) For suggestion 5, I suggest that the author use "at these light intensities" to make the statement more accurate.

Response:

Thank you for your valuble comment. We have addressed your suggestion in the revised manuscript, with the change highlighted in gray. Lines 126, 135, 150, 151, 161, and 282 have been changed according to your suggestion. The tracked changes for these sections are also visible in the marked-up manuscript

Re: Spectrum03782-25R3 (Changes in cyanobacterial growth and photosynthetic pigment under different conditions)

Dear Dr. Maryam Ameri:

Your manuscript has been accepted, and I am forwarding it to the ASM production staff for publication. Your paper will first be checked to make sure all elements meet the technical requirements. ASM staff will contact you if anything needs to be revised before copyediting and production can begin. Otherwise, you will be notified when your proofs are ready to be viewed.

Data Availability: ASM policy requires that data be available to the public upon online posting of the article, so please verify all links to sequence records, if present, and make sure that each number retrieves the full record of the data. If a new accession number is not linked or a link is broken, provide production staff with the correct URL for the record. If the accession numbers for new data are not publicly accessible before the expected online posting of the article, publication may be delayed; please contact ASM production staff immediately with the expected release date.

Publication Fees: For information on publication fees and which article types have charges, please visit our website. We have partnered with Copyright Clearance Center (CCC) to collect author charges. If fees apply to your paper, you will receive a message from no-reply@copyright.com with further instructions. For questions related to paying charges through RightsLink, please contact CCC at ASM_Support@copyright.com or toll free at +1-877-622-5543. CCC makes every attempt to respond to all emails within 24 hours.

ASM Membership: Corresponding authors may join or renew ASM membership to obtain discounts on publication fees. Need to upgrade your membership level? Please contact Customer Service at Service@asmusa.org.

Embargo Policy: A press release may be issued as soon as the manuscript is posted on the Spectrum Latest Articles webpage. The corresponding author will receive an email with the subject line "ASM Journals Author Services Notification" when the article is available online.

The ASM Journals program strives for constant improvement in our submission and publication process. Please tell us how we can improve your experience by taking this quick Author Survey.

Thank you for submitting your paper to Spectrum.

Sincerely,
Pedro Nuno Leão
Editor
Microbiology Spectrum
